# Genetic Analysis of Histamine Signaling in Larval Zebrafish Sleep

**DOI:** 10.1523/ENEURO.0286-16.2017

**Published:** 2017-03-02

**Authors:** Audrey Chen, Chanpreet Singh, Grigorios Oikonomou, David A. Prober

**Affiliations:** 1 Division of Biology and Biological Engineering, California Institute of Technology, Pasadena, CA 91125

**Keywords:** genetics, histamine, hypocretin, sleep, wake

## Abstract

Pharmacological studies in mammals and zebrafish suggest that histamine plays an important role in promoting arousal. However, genetic studies using rodents with disrupted histamine synthesis or signaling have revealed only subtle or no sleep/wake phenotypes. Studies of histamine function in mammalian arousal are complicated by its production in cells of the immune system and its roles in humoral and cellular immunity, which can have profound effects on sleep/wake states. To avoid this potential confound, we used genetics to explore the role of histamine in regulating sleep in zebrafish, a diurnal vertebrate in which histamine production is restricted to neurons in the brain. Similar to rodent genetic studies, we found that zebrafish that lack histamine due to mutation of histidine decarboxylase (*hdc*) exhibit largely normal sleep/wake behaviors. Zebrafish containing predicted null mutations in several histamine receptors also lack robust sleep/wake phenotypes, although we are unable to verify that these mutants are completely nonfunctional. Consistent with some rodent studies, we found that arousal induced by overexpression of the neuropeptide hypocretin (Hcrt) or by stimulation of *hcrt*-expressing neurons is not blocked in *hdc* or *hrh1* mutants. We also found that the number of *hcrt*-expressing or histaminergic neurons is unaffected in animals that lack histamine or Hcrt signaling, respectively. Thus, while acute pharmacological manipulation of histamine signaling has been shown to have profound effects on zebrafish and mammalian sleep, our results suggest that chronic loss of histamine signaling due to genetic mutations has only subtle effects on sleep in zebrafish, similar to rodents.

## Significance Statement

Based on pharmacological studies in several model organisms, histamine is thought to be a key arousal-promoting neuromodulator. However, genetic studies in rodents have reported only subtle phenotypes. Rodent studies are complicated by involvement of histamine in regulating the immune system, which itself affects sleep. In zebrafish, histamine production is restricted to neurons in the brain, thus allowing study of histamine function in arousal without confounding effects of abnormal immune system function. We show that zebrafish lacking histamine synthesis have largely normal sleep/wake behaviors, as do histamine receptor mutants, although we lack tools to verify that the receptor mutants are nonfunctional. These results suggest that genetic loss of histamine signaling has little effect on sleep/wake behaviors in zebrafish, similar to rodents.

## Introduction

Sleep is an essential and evolutionarily conserved behavioral state whose regulation remains poorly understood. Histamine, which is thought to promote arousal, is synthesized by histidine decarboxylase (Hdc), which is expressed in the hypothalamic tuberomammillary nucleus (TMN) (Thakkar, 2011). In mammals, histamine activates four receptor subtypes (Seifert et al., 2013). Histamine H1 receptor (Hrh1) is expressed widely in the brain, particularly in regions implicated in neuroendocrine, behavioral, and nutritional state control (Panula et al., 2015). Hrh2 is also expressed in several brain regions, as well as in peripheral tissues where it regulates gastric acid secretion, heart function, and the immune system (Panula et al., 2015). Hrh3 can act both as an autoreceptor that inhibits the synthesis and release of histamine and as a heteroreceptor that inhibits the release of other neurotransmitters (Schlicker et al., 1994; Morisset et al., 2000; Arrang et al., 2007; Haas et al., 2008; Samaranayake et al., 2016). In humans, Hrh3 is strongly expressed in the basal ganglia, hippocampus, and cortical areas (Martinez-Mir et al., 1990). Hrh4 is exclusively expressed in the periphery, where it functions in mast cells, eosinophils and T cells to mediate inflammatory, itch, and immune responses (de Esch et al., 2005).

Several studies have suggested that histamine promotes wakefulness in mice and *Drosophila*. For example, animals treated with Hdc inhibitors or Hrh1 antagonists exhibit sedation (Roth et al., 1987; Thakkar, 2011). However, mutants with disrupted histamine synthesis or signaling exhibit remarkably normal sleep/wake behaviors, primarily exhibiting subtle defects in wakefulness when high vigilance is required (Inoue et al., 1996; Kubota et al., 2002; Parmentier et al., 2002; Abe et al., 2004; Anaclet et al., 2009; Thakkar, 2011). The basis of the discrepancy between genetic and pharmacological manipulations remains unclear, although similar discrepancies have been reported for other sleep regulatory systems. For example, drugs that affect signaling via adenosine or noradrenergic receptors have significant effects on sleep, whereas mutations that affect adenosine or noradrenergic signaling result in only subtle phenotypes, in rodents (Berridge et al., 2012; Brown et al., 2012). Studying the role of histamine in mammalian arousal is complicated because, in addition to its neurologic functions, it also plays a central role in humoral and cellular immunity (Haas et al., 2008; Jutel et al., 2009). The zebrafish has recently emerged as a useful model organism to study sleep (Chiu and Prober, 2013) and may be particularly appropriate to explore the role of histamine in arousal, because histamine production is restricted to the TMN (Da'as et al., 2011) and the zebrafish genome appears to lack an ortholog of the peripherally expressed Hrh4 (Peitsaro et al., 2007). Furthermore, with only 10-12 histaminergic neurons, zebrafish larvae provide a simpler model to explore the role of these neurons in sleep (Sundvik et al., 2011). Previous studies showed that Hrh1 antagonists produce similar robust effects on zebrafish sleep as in mammals (Peitsaro et al., 2007; Renier et al., 2007; Rihel et al., 2010; Sundvik et al., 2011), but genetic manipulation of histamine signaling in zebrafish has not been reported.

To address the role of histamine in regulating sleep/wake states in a vertebrate animal that lacks histamine in the immune system, we generated zebrafish containing predicted null mutations in *hdc* and in histamine receptors. Similar to rodent *hdc* and *hrh* mutants (Inoue et al., 1996; Yanai et al., 1998; Parmentier et al., 2002; Abe et al., 2004; Anaclet et al., 2009), we found that sleep/wake states are largely normal in each larval zebrafish mutant. In contrast to one rodent report (Huang et al., 2001), but consistent with others (Carter et al., 2009; Hondo et al., 2010), we found that histamine is not required for arousal induced by overexpression of the neuropeptide hypocretin (Hcrt) or by stimulation of *hcrt*-expressing neurons in zebrafish larvae. Finally, in contrast to previous morpholino and pharmacology studies (Sundvik et al., 2011), we found that genetic blockage of Hcrt or histamine signaling does not affect the number of histaminergic or *hcrt*-expressing neurons, respectively. Together with previously described effects of pharmacological manipulation of histamine signaling in zebrafish (Peitsaro et al., 2007; Renier et al., 2007; Rihel et al., 2010; Sundvik et al., 2011), our results suggest that the role of histamine in regulating sleep/wake states is largely similar in zebrafish and mammals.

## Materials and Methods

### Zebrafish genetics

All animal procedures followed standard protocols (Westerfield, 2000) in accordance with the California Institute of Technology Institutional Animal Care and Use Committee guidelines. For most experiments, heterozygous adults were crossed to obtain homozygous mutant, heterozygous mutant and wild-type (WT) sibling larvae, which were genotyped after imaging or behavioral experiments. In cases where two or more mutants and/or transgenic animals were compared, we mated heterozygous adults to homozygous adults to reduce the number of genotypes present in their progeny and, thus, increase the number of animals of each genotype in the experiment. This was necessary to achieve sufficient animals of each genotype to obtain statistically robust data. This comparison is reasonable because heterozygous mutant animals did not exhibit phenotypes compared with their WT siblings. All experiments were performed using zebrafish larvae before the onset of sexual differentiation. *hdc* (RRID: ZDB-GENE-080102-5) mutant: TALEN binding sites were 5’-TCACTGCTGGGAGACA-3’ and 5’-TGAAGCCGAGGCAGTT-3’. *hdc* mutant d10 contains a 10-bp deletion (TGCTGGCAGA) after nucleotide 277 of the open reading frame. The mutation results in a change in reading frame after amino acid 92 and a premature stop codon after amino acid 164, compared with 608 amino acids for the WT protein. The predicted mutant protein lacks conserved residues that are required for function of the human *hdc* ortholog (Komori et al., 2012). *hdc* mutants were genotyped using the primers 5’-TACCCAGGTGAAGCCGAG-3’ and 5’-GCTGCAGTTCTGCTGTGTGT-3’, followed by digest with BsaHI (New England Biolabs), which cuts the 144-bp WT PCR product into 114 and 30 bp. *hrh1* (RRID: ZDB-GENE-070531-3) mutant: the *hrh1 hu3427* mutant was generated by the Zebrafish Mutation Project (Kettleborough et al., 2013) and contains an A/T nonsense mutation at nucleotide 1366 of the open reading frame, which is predicted to generate a 456-amino acid protein compared with the 534-amino acid WT protein. The mutant protein lacks two transmembrane domains and should thus be nonfunctional. *hrh1* mutants were genotyped using the primers 5’-TCCGCTGGACGCTAGTATTG-3’ and 5’-AGCCCAGCTGGCGCGCCGCTTTCCTCTCTT-3’, followed by digest with DdeI (New England Biolabs), which cuts the 125-bp mutant PCR product into 95 and 30 bp. *hrh2a* (RRID: ZDB-GENE-070531-4) mutant: TALEN binding sites were 5’-TCATCCTGCTCACTGTAA-3’ and 5’-TAGCATACACAGCCAGAC-3’. *hrh2a* mutant d10 contains a 10-bp deletion (AATATTCTGG) after nucleotide 63 of the open reading frame. The mutation results in a change in reading frame after amino acid 21 and a premature stop codon after amino acid 42, compared with 369 amino acids for the WT protein. The predicted mutant protein lacks six transmembrane domains and should thus be nonfunctional. *hrh2a* mutants were genotyped using the primers 5’-CTTTAGCTGTGACGCTCTCC-3’ and 5’-GCTAGCGAAACGATGAAGCA-3’, which produces a 124-bp PCR product for WT and a 114-bp product for the mutant. *hrh2b* (RRID: ZDB-GENE-070928-20) mutant: TALEN binding sites were 5’-TGACAGACCTACTTCT-3’ and 5’-TCCAGCATGGCAGAAAGT-3’. *hrh2b* mutant d8 contains an 8-bp deletion (TTGCTAGT) after nucleotide 162 of the open reading frame. The mutation results in a change in reading frame after amino acid 54 and a premature stop codon after amino acid 96, compared with 335 amino acids for the WT protein. The predicted mutant protein lacks six transmembrane domains and should thus be nonfunctional. *hrh2b* mutants were genotyped using 5’-CTGGTTTGTATGGCCGTGG-3’ and 5’-TTTCCATTGCGCAGTTCCAG-3’, which produces a 140-bp PCR product for WT and 132 bp for the mutant. *hrh3* (RRID: ZDB-GENE-040724-204) mutant: ZFN binding sites were 5’-TCCGTGGCG-3’ and 5’-GCAGTCCTC-3’. *hrh3* mutant d4 contains a 4-bp deletion (GTGG) after nucleotide 1022 of the open reading frame. The mutation results in a change in reading frame after amino acid 341 and a premature stop codon after amino acid 372, compared with 473 amino acids for the WT protein. The predicted mutant protein lacks two transmembrane domains and should thus be nonfunctional. *hrh3* mutants were genotyped using the primers 5’-GAAACGGTTGGCTAGACTGG-3’, 5’-CTTGCCTCCTCTGCAGAA-3’, and 5’-TGGCTTCAACCGCTAAAGTG-3’, which generate one band for WT (206 bp), two bands for homozygous mutant (202 and 123 bp), and three bands for heterozygous mutant (206, 202, and 123 bp).

Sequence alignments were performed using MegAlign Pro (DNASTAR).

The *hcrtr hu2098* mutant line (RRID: ZDB-ALT-070427-14) was generated by the Zebrafish Mutation Project (Kettleborough et al., 2013) and has previously been described (Yokogawa et al., 2007). *hcrtr* mutants were genotyped using the primers 5’-CCACCCGCTAAAATTCAAAAGCACTGCTAAC-3’ and 5’-CATCACAGACGGTGAACAGG-3’, followed by digest with DdeI, which cuts the 170-bp mutant PCR product into 140 and 30 bp.

The *Tg(hsp:Hcrt)* line (RRID: ZDB-TGCONSTRCT-070228-2) has previously been described (Prober et al., 2006). Transgenic animals were identified using the primers 5’-CGGGACCACCATGGACT-3’ and 5’-GGTTTGTCCAAACTCATCAATGT-3’, which generate a 470-bp PCR product.

*Tg(hcrt:ReaChR-mCitrine)*: the 1-kb zebrafish *hcrt* promoter (Faraco et al., 2006) was amplified using the primers 5’-ATAATAAATAAATCTGATGGGGTTTT-3’ and 5’-GAGTTTAGCTTCTGTCCCCTG-3’, and subcloned 5’ to a transgene encoding the channelrhodopsin-2 variant ReaChR (Lin et al., 2013) fused to mCitrine, in a plasmid containing flanking Tol2 transposase sequences, using Gibson assembly. Stable transgenic lines were generated using the Tol2 transposase method (Asakawa and Kawakami, 2009). Transgenic animals were identified using fluorescence or by PCR using the primers: 5’-CACGAGAGAATGCTGTTCCA-3’ and 5’-CCATGGTGCGTTTGCTATAA-3’, which generate a 431-bp product.

### Histamine ELISA

Adult fish were anesthetized in 0.2% tricaine and euthanized in ice water for 15 min before dissection in chilled PBS. Triplicate samples containing five adult brains each were homogenized in 160 µL of 0.2 N perchloric acid. Homogenate was spun at 4ºC at 10 × 10^3^ g for 5 min and applied to a 0.45-µm filter (UFC30HV00; Millipore). Filtrate was centrifuged at 4ºC at 10 × 10^3^g for 15 min. A total of 150 µL of the supernatant was collected and neutralized by adding 150-µL 1 M K_2_B_4_O_7_, pH 8.0. Histamine levels were assayed using a histamine ELISA kit (IM2015; Immunotech) according to the manufacturer’s instructions. Absorbance was read at 405 nm using a plate reader (Infinite M200Pro; Tecan). A calibration curve was generated by fitting absorbance values of manufacturer-provided standards to a 4-parameter logistic curve. In some cases, histamine readings of *hdc*−/− samples were lower than the 0 mM standard and were rounded up to 0 mM.

### Immunohistochemistry (IHC)

Five days postfertilization (dpf) larvae were fixed with freshly made, chilled 4% N-Ethyl-N′-(3-dimethylaminopropyl) carbodiimide hydrochloride (EDAC), 0.1% paraformaldehyde (PFA) in 0.1 M PBS for 16 h at 4ºC and then washed 3 × 5 min in 0.25% Triton X-100 in PBS (PBTx). Brains were manually dissected and blocked for 2 h at room temperature in 1% normal goat serum/1% DMSO/PBTx. For histamine IHC, brains were incubated in 1:10,000 rabbit antihistamine (AB5885, Millipore) in blocking solution overnight for three nights (∼70 h) at 4ºC with gentle agitation. Brains were washed 10 × 15 min with 1% DMSO/PBTx and incubated in 1:500 goat anti-rabbit Alexa Fluor 488 in blocking solution overnight at 4ºC. Samples were washed 10 × 15 min in 1% DMSO/PBTx, once in PBS and equilibrated in 50% glycerol/PBS. Samples were mounted in 50% glycerol/PBS. Images were acquired using an LD LCI Plan-Apochromat 25x Imm Corr DIC objective (0.8 NA; Zeiss) and a Zeiss 780 confocal microscope. Larval progeny of an +/− incross were used, and animals were genotyped by PCR after imaging. To verify specific ReaChR-mCitrine expression in *Tg(hcrt:ReaChR-mCitrine)* larvae, IHC and imaging were performed as described above using rabbit anti-orexin A (AB3704, 1:500; Millipore) and chicken anti-GFP (GFP-1020, 1:400; AvesLabs). Alexa Fluor secondary antibodies were used (1:500 Alexa Fluor 568 anti-rabbit and 1:600 Alexa Fluor 488 anti-chicken, Life Technologies).

### *In situ* hybridization (ISH)

Samples were fixed in 4% PFA/PBS for 16 h at room temperature. ISH was performed using digoxygenin (DIG)-labeled antisense riboprobes (DIG RNA Labeling kit; Roche) as previously described (Thisse and Thisse, 2008). Probes specific for *hcrt* (hcrt; Prober et al., 2006) and *hdc* (hdc; Eriksson et al., 1998) have been described. Images were acquired using a Zeiss AxioImager M1 microscope. Larval progeny of an +/− incross were used, and animals were genotyped by PCR after imaging.

### Sleep-wake analysis

Mammalian sleep is typically monitored using electrophysiology, but this can be difficult to perform in nonmammalian systems. In these cases, behavioral criteria can be used to define sleep states (Campbell and Tobler, 1984; Allada and Siegel, 2008). First, sleep is usually observed as locomotor quiescence that occurs during specific periods of the circadian cycle. Second, animals exhibit an increased arousal threshold during sleep, which distinguishes sleep from quiet wakefulness. Third, sleep is rapidly reversible, and sleeping animals can be aroused by strong stimuli, thus distinguishing sleep from paralysis or coma. Fourth, sleep is controlled by a homeostatic system, which can be demonstrated by an increased need for sleep following sleep deprivation. Using these criteria, we and others (Prober et al., 2006; Elbaz et al., 2012) have shown that 1 or minute of inactivity corresponds to a sleep state in zebrafish larvae. Waking activity is defined as the amount of locomotor activity not including sleep periods. Sleep latency is defined as the amount of time between lights on or off and the first sleep bout.

Larvae were raised on a 14/10 h light/dark cycle at 28.5ºC with lights on at 9 A.M. and off at 11 P.M. Individual larvae were placed into each well of a 96-well plate (7701-1651; Whatman) containing E3 embryo medium (5 mM NaCl, 0.17 mM KCl, 0.33 mM CaCl_2_, and 0.33 mM MgSO_4_, pH 7.4) at 4 dpf in the evening. Plates were sealed with an optical adhesive film (4311971; Applied Biosystems) to prevent evaporation. We have not observed adverse effects of sealing the plate on animal health or behavior, and rather we observe more robust behaviors that last longer in sealed plates. In nonsealed plates, there is significant evaporation of water from each well each day, and thus, changes in ion concentrations in the water. Thus, although we add fresh water to each well every day in nonsealed plates, there are still daily fluctuations in ion concentrations that likely lead to less robust health and behavior. The sealing process introduces air bubbles in some wells, which are discarded from analysis. Locomotor activity was monitored using a videotracking system with a Dinion one-third inch Monochrome camera (Dragonfly 2; Point Gray) fitted with a variable-focus megapixel lens (M5018-MP; Computar) and infrared filter. The movement of each larva was captured at 15 Hz and recorded using the quantization mode with 1-min integration time bins. The 96-well plate and camera were housed inside a custom-built Zebrabox (Viewpoint Life Sciences) that was continuously illuminated with infrared lights. The 96-well plate was housed in a chamber filled with recirculating water to maintain a constant temperature of 28.5ºC. The parameters used for movement detection were as follows: detection threshold, 15; burst, 29; freeze, 3. These values are used by the software to detect and score movements. They do not have standard units and must be set empirically. Data were analyzed using custom Perl and Matlab (Mathworks) scripts, and statistical tests were performed using Prism (GraphPad) and Matlab.

To assay behavioral responses to an environmental challenge, we exposed *hdc* and *hrh1* mutants to alternating periods of light and darkness. Larvae were transferred to a 96-well plate at 5 dpf in the afternoon and were exposed to alternating 1-h periods of light and darkness for 24 h starting at 5 P.M. We compared the brief increases in locomotor activity that are induced by light onset and offset (Prober et al., 2006; Burgess and Granato, 2007) and also quantified total locomotor activity during each light and dark period, with data collected in 5-s bins. Because light offset induces an increase in locomotor activity that lasts ∼10 min, we excluded these data from dark period locomotor activity quantification. For the analysis presented in the paper, we quantified locomotor activity during light and dark periods averaged for five light/dark cycles at night because the behavioral response to light onset is most apparent at night, and because it is difficult to compare features of behavioral traces that encompass longer time periods. In addition, locomotor activity levels are higher for all genotypes during the day than at night. As a result, combining data from the day and night risked obscuring subtle mutant phenotypes, although data for light and dark periods averaged over the entire 24-h experiment yielded results similar to those shown in Figure 6 (data not shown).

For Hcrt overexpression experiments, videotracker analysis was initiated at 4 dpf. During the afternoon of 6 dpf, the 96-well plate was transferred to a 37ºC water bath for 1 h to induce Hcrt overexpression. Preheat shock behavior was calculated from day 5 and night 5, and postheat shock behavior was calculated from night 6 and day 7. Two-way ANOVA was used to examine effects of heat shock (pre vs post) and genotype on behavior.

### Mechano-acoustic stimulus assay

This assay was performed, and data were analyzed as described previously (Singh et al., 2015). The videotracking system was modified by adding an Arduino based automated driver to control two solenoids (28P-I-12; Guardian Electric) that delivered a tap to a 96-well plate containing larvae. This setup allowed us to drive the solenoids with voltage ranging from 0 to 20 V over a range of 4095 settings (from 0.01 to 40.95). We used taps ranging from a power setting of 1-36.31. Taps of 14 different intensities were applied in a random order from 12:30 to 7:30 A.M. during the fifth day or night of development with an intertrial-interval of 1 min. Previous studies showed that a 15-s interval between repetitive stimuli is sufficient to prevent behavioral habituation (Burgess and Granato, 2007; Woods et al., 2014). The background probability of movement was calculated by identifying for each genotype the fraction of larvae that moved 5 s before all stimuli delivered during an experiment (14 different tap powers × 30 trials per experiment = 420 data points per larva; average background movement). This value was subtracted from the average response fraction value for each tap event (corrected response = average response – average background movement). Tapping experiments with a 5-min intertrial interval were performed from 12:30 to 9:00 A.M. using a single tap intensity (3.02) to assess the response of sleeping animals to the stimulus. The response of larvae to the stimuli was monitored using the videotracking software and was analyzed using Matlab (The Mathworks) and Excel (Microsoft). Curve fitting was performed using the Variable Slope log(dose) response curve-fitting module of Prism (GraphPad).

### Optogenetic assay

This assay was performed, and data were analyzed as described (Singh et al., 2015). The videotracking system was modified to include an array of three sets of blue LEDs (470 nm, MR-B0040-10S, Luxeon V-star) mounted 15 cm above and 7 cm away from the center of the 96-well plate to ensure uniform illumination. The LEDs were controlled using a custom built driver and software written in BASIC stamp editor. A power meter (1098293, Laser-check) was used before each experiment to verify uniform light intensity (∼450 µW at the surface of the 96-well plate). During the afternoon of the fifth day of development, single larvae were placed into each well of a 96-well plate as described above and placed in the videotracker in the dark for 8 h. Larvae were then exposed to blue light for 30 min, starting at 1 A.M. Three trials were performed during the night, with an intertrial interval of 3 h. Total activity for each larva was monitored for 30 min before and after light onset, with data collected in 10-s bins. Light onset caused a short burst of locomotor activity lasting for ∼30 s for all genotypes, so data obtained during the minute before and after light onset were excluded from analysis. A large burst of locomotor activity was also observed for all genotypes when the lights were turned off after the 30-min illumination period. These data were excluded from analysis and are not shown in the figures. Data from three independent experiments were merged before analysis. The total amount of locomotor activity of each larva during the 30 min of light exposure, excluding the minute after light onset, was divided by the average baseline locomotor activity for all larvae of the same genotype. The baseline period was defined as 30 min before light onset, excluding the minute before light onset.

### Statistics

Data were analyzed using one-way ANOVA, two-way ANOVA, or *t* tests, as appropriate. Tukey’s HSD test was used in *post hoc* analyses that compared three or more genotypes. For mechano-acoustic experiments with 1-min intertrial intervals, statistical significance was assessed using the extra sum-of-squares *F* test. For mechano-acoustic experiments with 5-min intertrial intervals, statistical significance was assessed using one-way ANOVA.

## Results

### Generation of a zebrafish *hdc* mutant that lacks histamine

**Figure 1.**
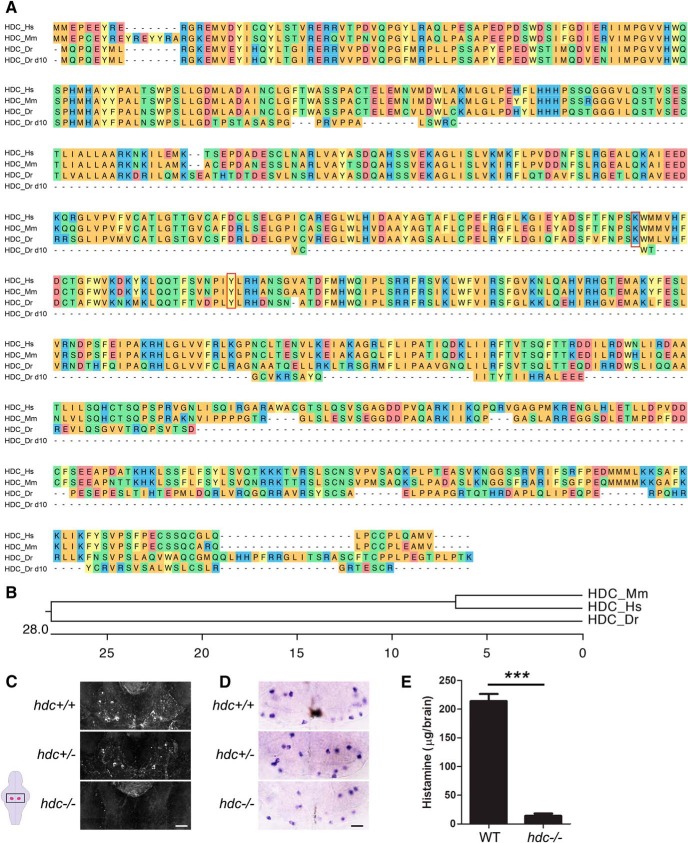
Characterization of *hdc*−/− zebrafish. ***A***, Alignment of Hdc protein sequences from human (Hs, ENSG00000140287), mouse (Mm, ENSMUSG00000027360), zebrafish WT (Dr, ENSDARG00000075454), and zebrafish 10-bp deletion mutant (Dr d10). The red box indicates an amino acid required for the activity of human Hdc (Komori et al., 2012a). Amino acids are colored to indicate residues with similar properties. ***B***, Phylogenetic tree of human, mouse, and zebrafish Hdc. Values indicate the number of amino acid substitutions per 100 residues. ***C***, ***D***, Ventral views of 5 dpf larval brains in which histaminergic neurons are labeled using IHC with a histamine-specific antibody (***C***) or ISH using an *hdc*-specific riboprobe (***D***) are shown for *hdc*+/+, *hdc*+/−, and *hdc*−/− siblings. Histamine is reduced in *hdc*+/− larvae and undetectable in *hdc*−/− larvae (***C***), but the number of *hdc*-expressing neurons is only slightly reduced in *hdc*−/− larvae compared with their *hdc*+/+ and *hdc*+/− siblings (***D***). Boxed region in schematic diagram (lower left) indicates region shown in (***C***, ***D***), with the TMN shaded magenta. Scale bars: 20 µm. ***E***, Histamine concentration assayed by ELISA from WT and *hdc*−/− adult brains. Little or no histamine was detected in *hdc*−/− fish. Histamine detected in *hdc*−/− animals is below the level of assay sensitivity and precision. ***p < 0.001 by Student’s *t* test.

Within the vertebrate central nervous system, histamine is produced exclusively in the TMN due to expression of hdc, which converts histidine to histamine (Schwartz et al., 1991). The zebrafish genome contains a single *hdc* ortholog, which encodes for a 595-amino acid protein (Fig. 1*A*). Zebrafish larvae have 10-15 *hdc*-expressing neurons per brain hemisphere (Sundvik and Panula, 2015), compared with ∼5000 in rodents (Ericson et al., 1987) and ∼60,000 in humans (Airaksinen et al., 1991). To explore the role of histamine in regulating sleep in zebrafish, we generated a *hdc* mutant using the TALEN method (Sander et al., 2011). We isolated an *hdc* mutant containing a 10-bp deletion, which results in a predicted 164-amino acid protein that lacks conserved residues required for function of the human *hdc* ortholog (Komori et al., 2012; Fig. 1*A*,*B*). To determine whether zebrafish *hdc* mutants produce histamine, we performed IHC using a histamine-specific antibody. At 5 dpf, we observed robust antibody labeling in 14.9 ± 0.8 cells (*n* = 10 larvae) in the TMN of *hdc*+/+ larvae (Fig. 1*C*). We observed a similar number of neurons in *hdc*+/− larvae (13.8 ± 1.3 cells, *n* = 13 larvae), although histamine levels were reduced compared with their *hdc*+/+ siblings (Fig. 1*C*). In contrast, histamine labeling was absent in *hdc*−/− larvae (0 cells, *n* = 6 larvae) (Fig. 1*C*). Using ISH, we observed that the level of *hdc* mRNA and the number of *hdc*-expressing neurons were similar for *hdc*+/+ (19.6 ± 0.7 cells, *n* = 17) and *hdc*+/− (17.7 ± 0.4 cells, *n* = 26) larvae (Fig. 1*D*). In *hdc*−/− larvae, *hdc* expression was slightly weaker and there were slightly fewer *hdc*-expressing cells (15.8 ± 0.9 cells, *n* = 17, *p* < 0.001 compared with *hdc*+/+ by Tukey’s HSD test), presumably due to nonsense-mediated mRNA decay (Isken and Maquat, 2007). These observations indicate that most TMN neurons are present in *hdc*−/− larvae, but they do not produce histamine. We also measured histamine levels by ELISA in adults, and found that *hdc*+/+ animals have 214 ± 12 µg histamine per brain (*n* = 15 animals), while little or no histamine was detected in their *hdc−/−* siblings (Fig. 1*E*). These results indicate that larval and adult *hdc*−/− zebrafish produce little or no histamine.

### *hdc* mutant zebrafish larvae exhibit largely normal sleep/wake behaviors

**Figure 2.**
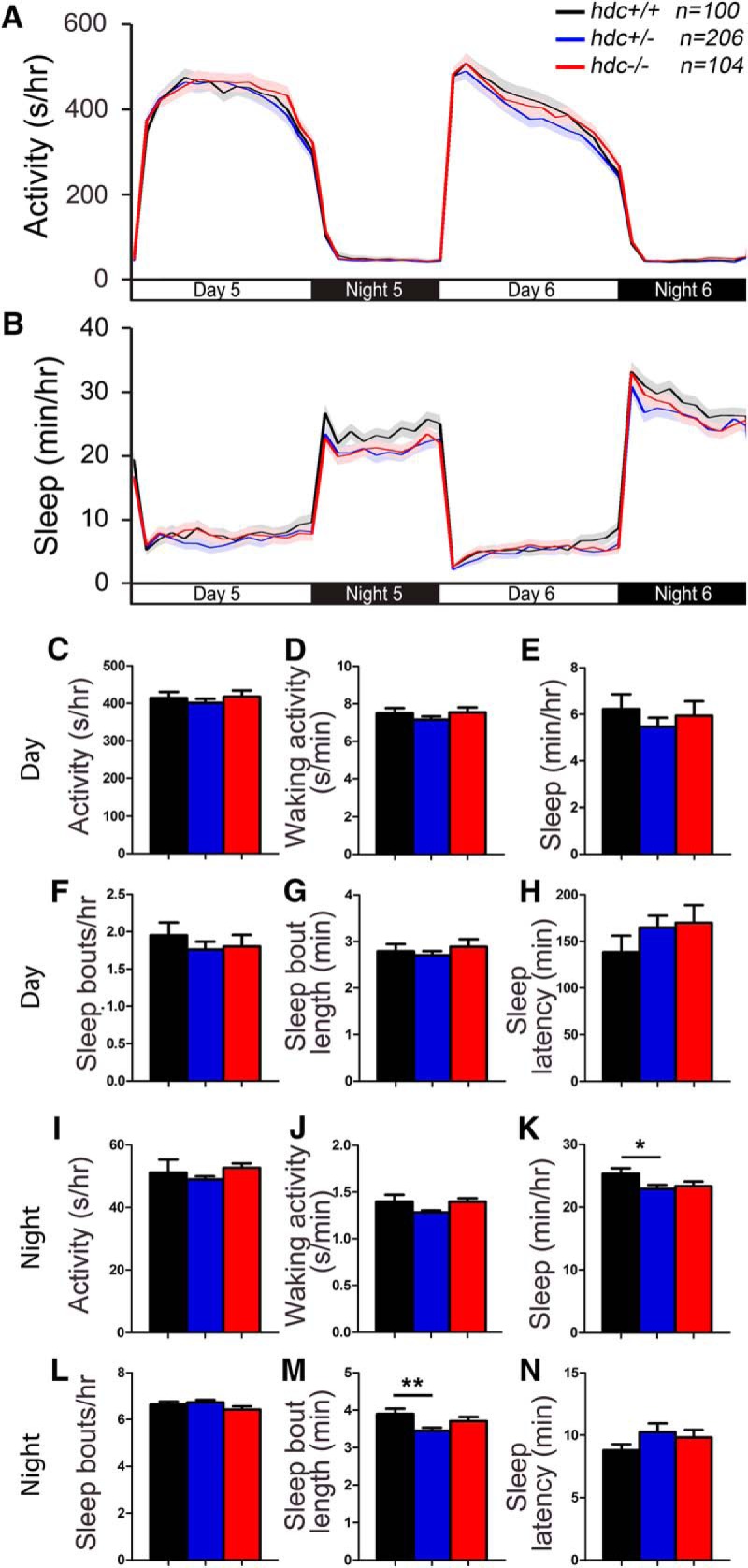
*hdc* mutant larvae exhibit normal sleep/wake behaviors. *hdc*−/− (red), *hdc*+/− (blue) and *hdc*+/+ (black) siblings exhibit similar amounts of all measured parameters, except that *hdc*+/− larvae sleep slightly less (***K***) and have slightly shorter sleep bouts (***M***) at night compared with their *hdc*+/+ siblings. Line and bar graphs represent the mean ± SEM for five experiments combined; *n* indicates the number of animals analyzed. *p < 0.05; **p < 0.01 for the indicated comparisons by one-way ANOVA with Tukey’s HSD test.

To determine whether histamine is required for normal larval zebrafish sleep/wake behaviors, we analyzed locomotor activity and sleep using a high-throughput behavioral assay (Prober et al., 2006). We observed that *hdc−/−* larvae exhibit similar levels of locomotor activity (Fig. 2*A*,*C*,*I*) and waking activity (Fig. 2*D*,*J*) as their *hdc*+/− and *hdc*+/+ siblings. Larvae of all three genotypes also exhibited similar total sleep amount during the day (Fig. 2*B*,*E*), as well as a similar number of sleep bouts (Fig. 2*F*,*L*) and sleep latency (Fig. 2*H*,*N*) during the day and night. *hdc*+/− animals exhibited slightly decreased sleep and sleep bout length at night compared with their *hdc*+/+ siblings (Fig. 2*B*,*K*,*M*), the latter of which is consistent with the fragmented sleep reported for *hdc*−/− mice (Parmentier et al., 2002). The significance of these effects is unclear because these measures were not significantly different for *hdc*−/− zebrafish compared with their *hdc*+/+ siblings. However, this discrepancy could be explained if compensatory mechanisms in *hdc*−/− animals rescue effects of reduced histamine levels that are observed in *hdc*+/− siblings (see discussion). Taken together, we conclude that zebrafish *hdc* mutants have largely normal total amounts of sleep and wakefulness in a 24-h period, similar to *hdc* knock-out mice (Parmentier et al., 2002). However, zebrafish *hdc−/−* larvae do not display decreased spontaneous activity or decreased sleep latency, as reported in some *hdc* knock-out mouse studies (Kubota et al., 2002; Parmentier et al., 2002; Abe et al., 2004; Anaclet et al., 2009).

**Figure 3.**
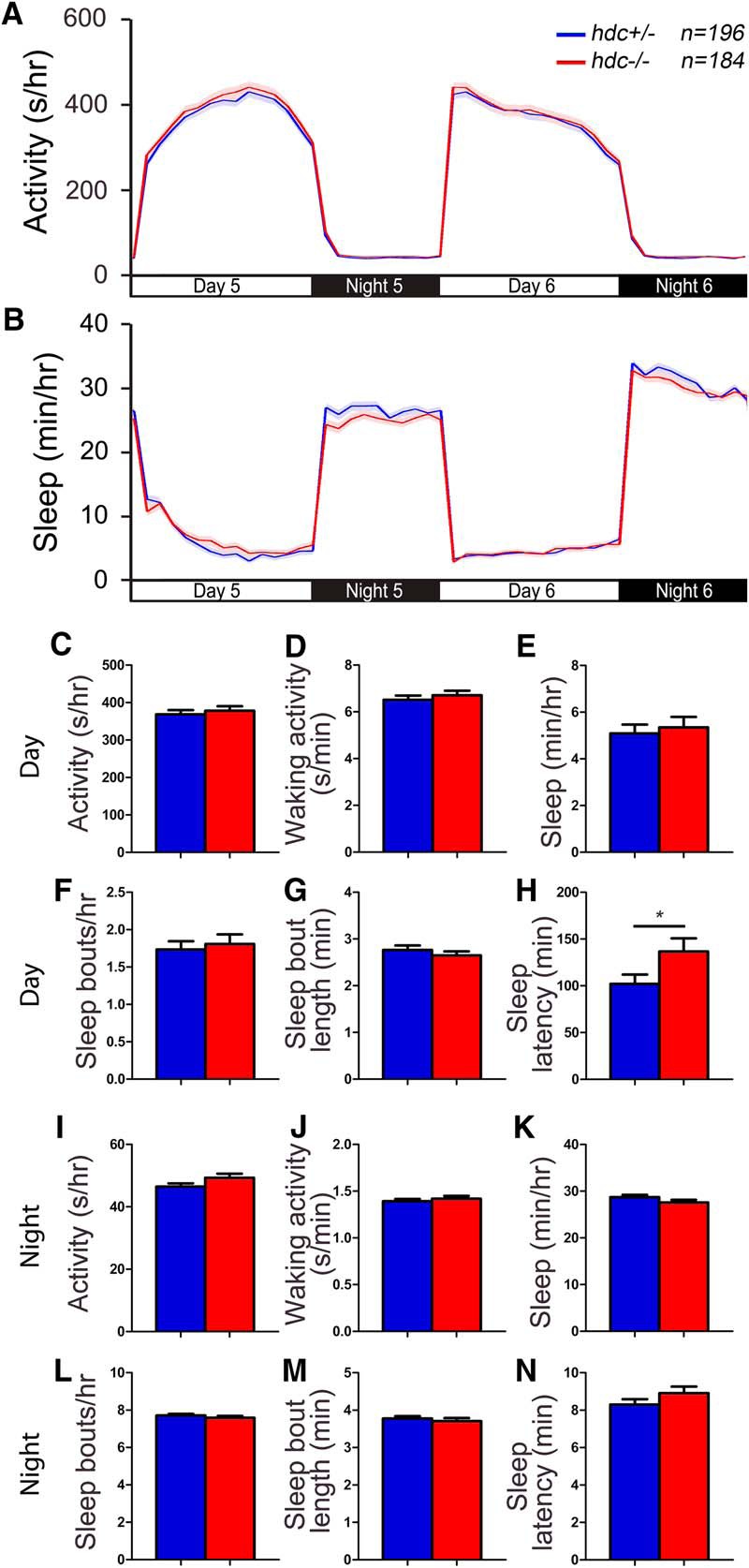
The absence of a larval *hdc* zygotic mutant behavioral phenotype is not due to rescue by maternal WT *hdc*. *hdc*−/− (red) and *hdc*+/− (blue) larvae that were generated by mating an *hdc*−/− female to an *hdc*+/− male fish do not show major changes in measured sleep/wake behaviors, except that *hdc*−/− larvae have a longer latency to first sleep bout during the day (***H***). Line and bar graphs represent the mean ± SEM for five experiments combined; *n* indicates the number of animals analyzed. *p < 0.05 for the indicated comparison by Student’s *t* test.

To test whether the absence of behavioral phenotypes in *hdc*−/− larvae is due to rescue by maternally deposited WT *hdc* mRNA, we mated *hdc*−/− females to *hdc*+/− males and examined the behavior of their *hdc*+/− and *hdc−/−* progeny. We again observed no phenotypes in *hdc−/−* larvae compared with their *hdc*+/− siblings (Fig. 3*A*–*G*,*I*–*N*), except that *hdc−/−* larvae had an increased latency to the first sleep episode following lights on in the morning (Fig. 3*H*), although this effect barely reached statistical significance. These results indicate that maternally contributed WT *hdc* mRNA may contribute modestly to daytime sleep latency, but otherwise does not account for the absence of sleep/wake phenotypes in zygotic *hdc−/−* larvae.

### *hrh1* mutant larvae do not show major changes in sleep/wake behaviors

While the *hdc* mutant data suggest that histamine is not required for normal sleep/wake behaviors in zebrafish larvae, histamine receptors exhibit constitutive ligand-independent signaling (Seifert et al., 2013), so we decided to explore potential roles for histamine receptors in larval zebrafish sleep. In mammals, histamine H1 receptor (Hrh1) is thought to mediate the effects of histamine on arousal because Hrh1 antagonists are sedating (Roth et al., 1987) and pretreatment with the Hrh1 antagonist pyrilamine blocks the arousing effect of histamine (Thakkar, 2011). *hrh1* knock-out mice, however, exhibit reduced locomotor activity only when challenged with a novel environment (Inoue et al., 1996; Yanai et al., 1998) and surprisingly show increased activity during the day (Inoue et al., 1996), but otherwise exhibit largely normal sleep/wake behaviors (Huang et al., 2006).

**Figure 4.**
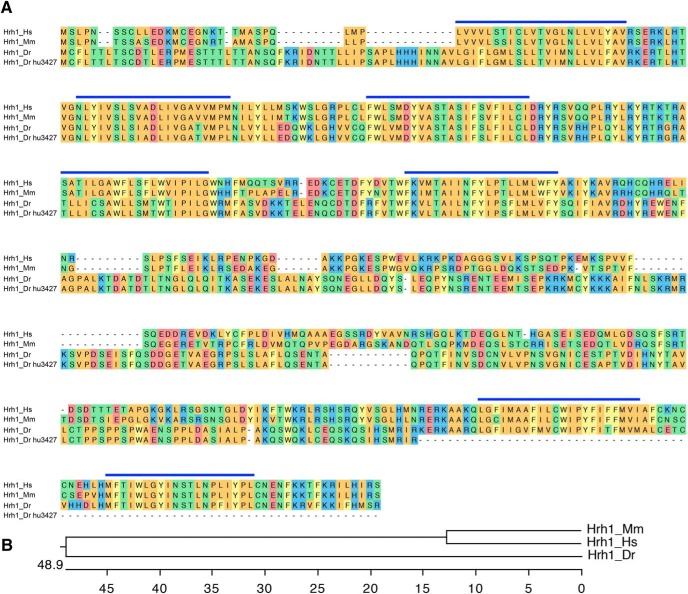
Alignment of Hrh1. ***A***, Hrh1 protein sequences from human (Hs, ENSG00000196639), mouse (Mm, ENSMUSG00000053004), zebrafish WT (Dr, ENSDARG00000052425), and zebrafish mutant (Dr hu3427). Blue lines indicate predicted transmembrane domains. Amino acids are colored to indicate residues with similar properties. ***B***, Phylogenetic tree of human, mouse, and zebrafish Hrh1. Values indicate the number of amino acid substitutions per 100 residues.

**Figure 5.**
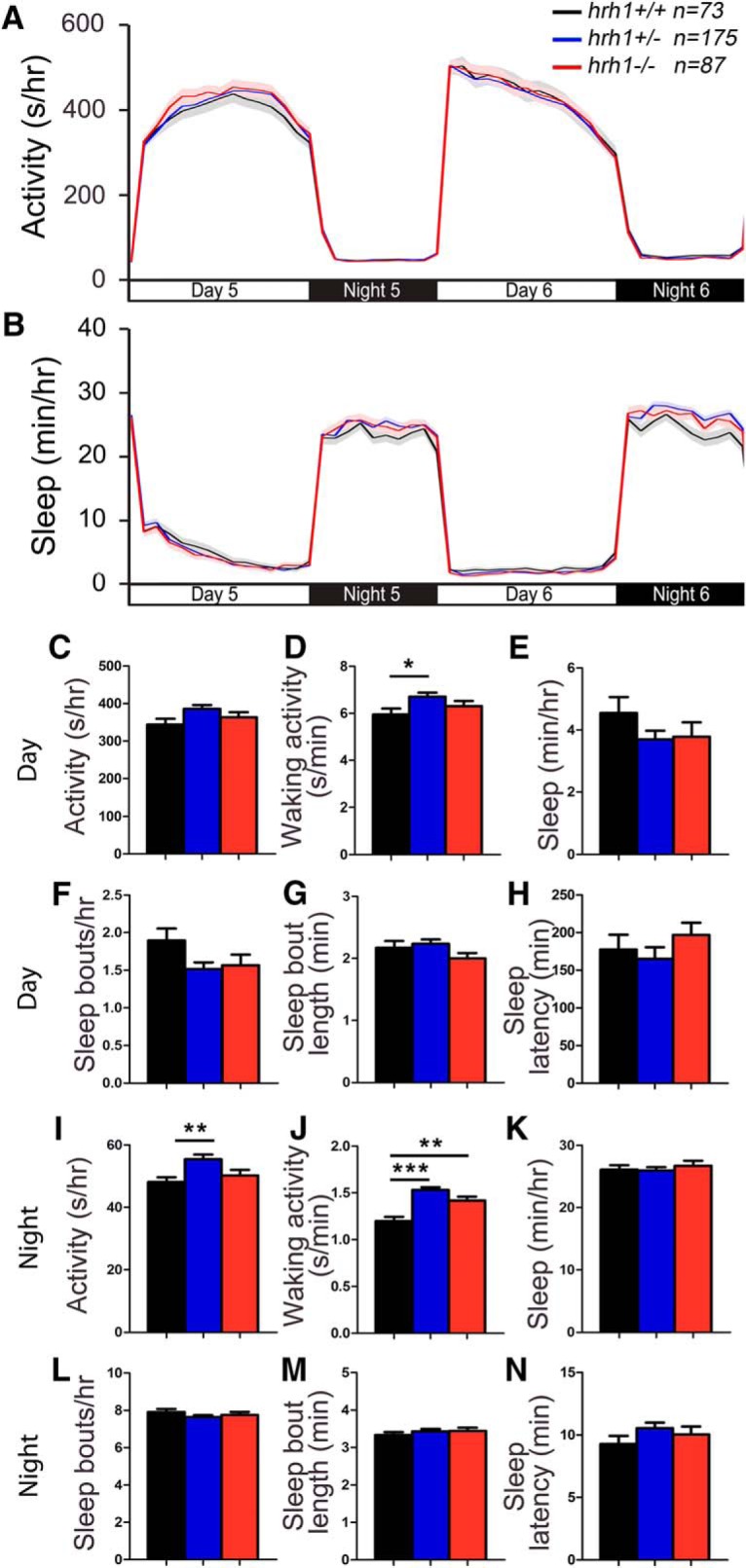
*hrh1* mutant larvae exhibit largely normal sleep/wake behaviors. *hrh1*−/− (red), *hrh1*+/− (blue), and *hrh1*+/+ (black) siblings exhibit similar amounts of all measured parameters, except that *hrh1*−/− *and hrh1*+/− larvae exhibit higher waking activity at night compared with *hrh1*+/+ siblings (***J***), and *hrh1*+/− larvae exhibit higher waking activity during the day (***D***) and higher activity at night (***I***) compared with their *hrh1*+/+ siblings. Line and bar graphs represent the mean ± SEM for four experiments combined; *n* indicates the number of animals analyzed. *p < 0.05; **p < 0.01; ***p < 0.001 for the indicated comparisons by one-way ANOVA with Tukey’s HSD test.

The zebrafish genome appears to contain a single Hrh1 ortholog that is 40% identical to human Hrh1 (Fig. 4; Peitsaro et al., 2007). Similar to mammals, *hrh1* is expressed in the ventral telencephalon, diencephalic and thalamic regions, and lateral hypothalamus in larval zebrafish (Sundvik et al., 2011). To determine whether Hrh1 is required for normal zebrafish sleep/wake states, we tested an *hrh1* mutant that contains a nonsense mutation and is predicted to generate a truncated protein that lacks two transmembrane domains, and should thus be nonfunctional (Fig. 4). *hrh1*−/− and *hrh1*+/− larvae exhibited slightly higher waking activity compared with their *hrh1*+/+ siblings at night (Fig. 5*J*), similar to *hrh1*−/− mice, which are hyperactive during the day, the rest period of these nocturnal animals (Inoue et al., 1996). *Hrh1*+/− larvae also showed increased waking activity during the day (Fig. 5*D*) and increased activity at night (Fig. 5*I*) compared with their *hrh1*+/+ siblings, although the significance of these effects is unclear because they were absent in *hrh1*−/− larvae. We conclude that *hrh1*−/− larvae lack major changes in sleep/wake behaviors compared with sibling controls, similar to *hrh1*−/− mice (Huang et al., 2006) and *hdc*−/− zebrafish (Figs. 2, 3).

### *hdc* and *hrh1* mutant larvae exhibit normal behavioral responses to environmental challenges

**Figure 6.**
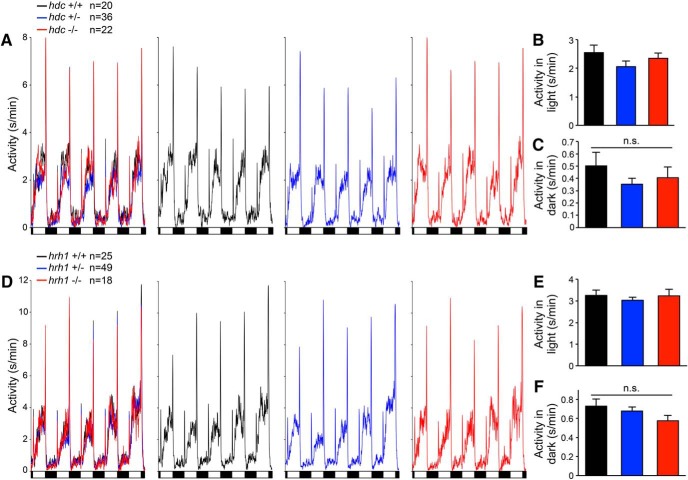
*hdc* and *hrh1* mutants exhibit normal behavioral responses to alternating periods of light and darkness. ***A***, ***D***, Line graphs represent the mean locomotor activity of *hdc*+/+ (black), *hdc*+/− (blue), and *hdc*−/− (red) larvae (***A***), or *hrh1*+/+ (black), *hrh1*+/− (blue), and *hrh1*−/− (red) larvae (***D***), plotted in a single graph containing all three genotypes (left) as well as in graphs showing individual genotypes to facilitate comparisons. Similar behavioral responses to light onset and offset are apparent for *hdc*−/− (***A***) and *hrh1*−/− (***D***) larvae compared with sibling controls. Black and white boxes indicate 1-h periods of light and darkness. ***B***, ***C***, ***E***, ***F***, Bar graphs represent the mean ± SEM locomotor activity during light and dark periods. There is no significant difference in the amount of locomotor activity during either light or dark periods for *hdc*−/− (***B***, ***C***) or *hrh1*−/− (***E***, ***F***) larvae compared with sibling controls. Data for five light/dark cycles at night is shown, but similar results are obtained when data are averaged over the entire 24-h experiment; *n* indicates the number of animals analyzed. n.s. = not significant (*p* > 0.05) for all comparisons by one-way ANOVA with Tukey’s HSD test.

**Figure 7.**
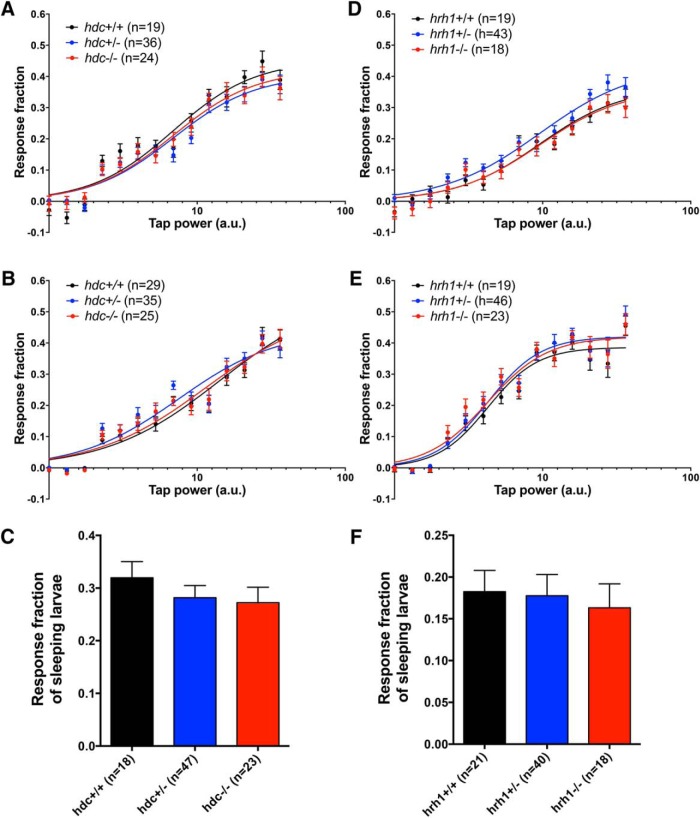
hdc and *hrh1* mutants exhibit normal behavioral responses to a mechano-acoustic stimulus. *hdc*+/+, *hdc*+/−, and *hdc*−/− larvae have the same response profile during the day (***A***) and night (***B***) (*p* > 0.05, extra sum-of-squares *F* test), and are equally likely to respond to tapping when asleep (***C***) (*p* > 0.05, one-way ANOVA). Similar results were obtained for *hrh1*+/+, *hrh1*+/−, and *hrh1*−/− larvae (***D–F***). Bar graphs represent the mean ± SEM; *n* indicates the number of animals analyzed.

To explore whether environmental challenges uncover sleep/wake defects in *hdc* and *hrh1* mutant zebrafish, as they do in rodents (Inoue et al., 1996; Yanai et al., 1998; Parmentier et al., 2002; Anaclet et al., 2009), we challenged animals using two paradigms. First, we exposed larvae to alternating 1-h periods of light and darkness for 24 h (Fig. 6). As previously shown, WT larvae exhibit a brief startle response to lights on, followed by a gradual increase in locomotor activity levels that reach a plateau, followed by a strong locomotor response when the lights are turned off (Prober et al., 2006; Burgess and Granato, 2007). We observed no significant differences in behavioral response to either light transition, or in total amount of locomotor activity during the light or dark periods, for *hdc*−/− or *hrh1*−/− animals compared with sibling controls (Fig. 6). This contrasts with a previous study, in which larvae injected with an *hdc*-specific morpholino, or treated with the Hdc inhibitor α-fluoromethylhistidine (α-FMH) or the Hrh1 antagonist pyrilamine, failed to respond to a lights off stimulus (Sundvik et al., 2011). Second, we subjected larvae to a mechano-acoustic stimulus of varying intensity every minute for 7.5 h during the day or night. We previously showed that larvae exhibit a brief startle response in a manner that depends on stimulus intensity and that does not habituate under these conditions. We observed no difference in the response of *hdc*−/− or *hrh1*−/− animals compared with sibling controls (Fig. 7*A*,*B*,*D*,*E*). We also performed a similar assay during the night using a 5-min intertrial interval and a single stimulus intensity (Fig. 7*C*,*F*). Since sleep in zebrafish larvae is defined as one or more minutes of inactivity, by allowing 5 min between trials, we could assess the response of specifically sleeping larvae and thus determine whether arousal from the sleep state is affected by mutation of *hdc* or *hrh1*. We found that sleeping mutant larvae and sibling controls were equally responsive to the stimulus. We conclude that *hdc* and *hrh1* mutants exhibit normal behavioral responses to these environmental perturbations, as well as normal sleep depth.

### *hrh2* mutant larvae lack sleep/wake phenotypes

**Figure 8.**
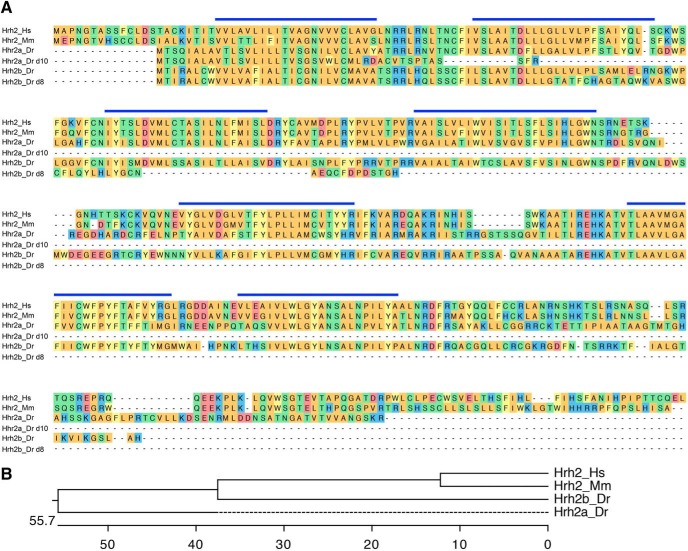
Alignment of Hrh2. ***A***, Hrh2 protein sequences from human (Hs, ENSG00000113749), mouse (Mm, ENSMUSG00000034987), zebrafish WT (Hrh2a_Dr, NM_001045338; Hrh2b_Dr, ENSDARG00000057479), and zebrafish mutant (Hrh2a_Dr d10; Hrh2b_Dr d8). Blue lines indicate predicted transmembrane domains. Amino acids are colored to indicate residues with similar properties. ***B***, Phylogenetic tree of human, mouse, and zebrafish Hrh2. Values indicate the number of amino acid substitutions per 100 residues.

**Figure 9.**
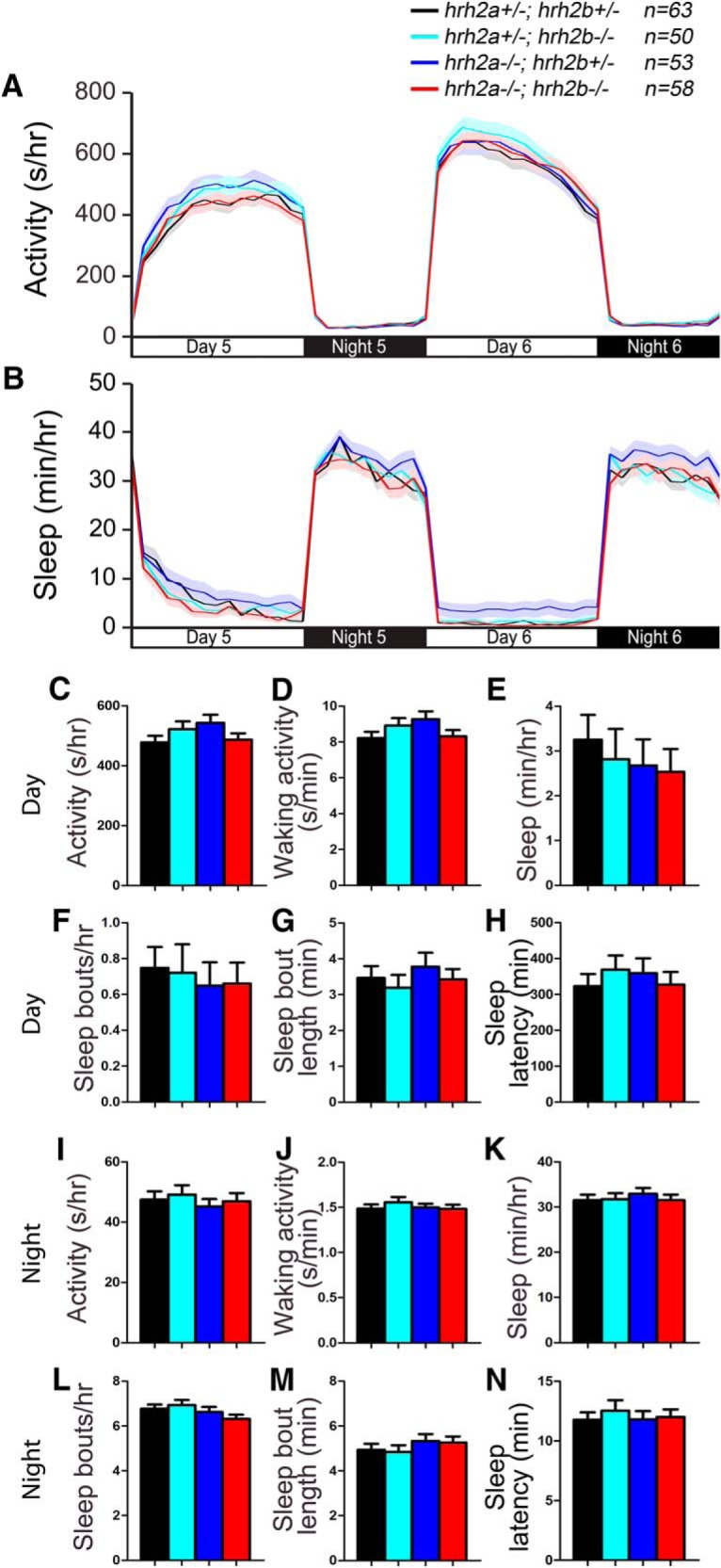
*hrh2* mutant larvae exhibit normal sleep/wake behaviors. *hrh2a*−/−*; hrh2b*−/− (red), *hrh2a*+/−*; hrh2b*+/− (black), *hrh2a*+/−*; hrh2b*−/− (cyan), and *hrh2a*−/−*; hrh2b*+/− (blue) larvae exhibit similar amounts of all measured sleep/wake parameters. Line and bar graphs represent the mean ± SEM for three experiments combined; *n* indicates the number of animals analyzed. *p* > 0.05 for all comparisons by one-way ANOVA with Tukey’s HSD test.

It is unclear whether Hrh2 signaling is involved in sleep/wake regulation. Systemic administration of Hrh2 agonists and antagonists has no effect on sleep or wake levels in nocturnal rodents (Monti et al., 1986; Monti et al., 1990), but application of an Hrh2 agonist in the preoptic region of the brain promotes wakefulness in cats (Lin et al., 1994; Lin et al., 1996). To determine whether signaling through Hrh2 is required for normal sleep/wake behaviors, we generated mutations in the two zebrafish *hrh2* paralogs, *hrh2a* and *hrh2b* (Fig. 8). Both mutants contain early stop codons and generate predicted proteins that lack several transmembrane domains and should thus be nonfunctional. To assay these mutants for sleep phenotypes, we compared the behavior of larvae generated from an incross of *hrh2a*+/−*; hrh2b*+/− animals. We found that the behavior of *hrh2a*−/−*; hrh2b*−/− larvae was indistinguishable from their *hrh2a*+/−*; hrh2b*+/− siblings (Fig. 9). Thus, mutation of zebrafish Hrh2 receptors does not result in detectable sleep/wake phenotypes.

### *hrh3* mutant larvae do not show major changes in sleep/wake behaviors

**Figure 10.**
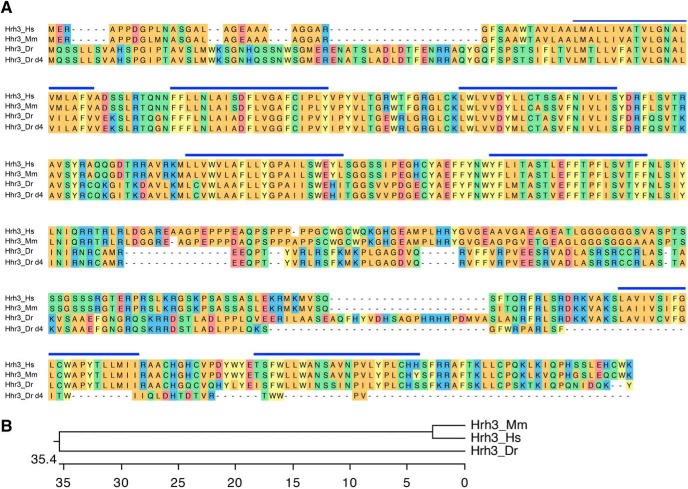
Alignment of Hrh3. ***A***, Hrh3 protein sequences from human (Hs, ENSG00000101180), mouse (Mm, ENSMUSG00000039059), zebrafish WT (Dr, ENSDARG00000035942), and zebrafish mutant (Dr d4). Blue lines indicate predicted transmembrane domains. Amino acids are colored to indicate residues with similar properties. ***B***, Phylogenetic tree of human, mouse, and zebrafish Hrh3. Values indicate the number of amino acid substitutions per 100 residues.

**Figure 11.**
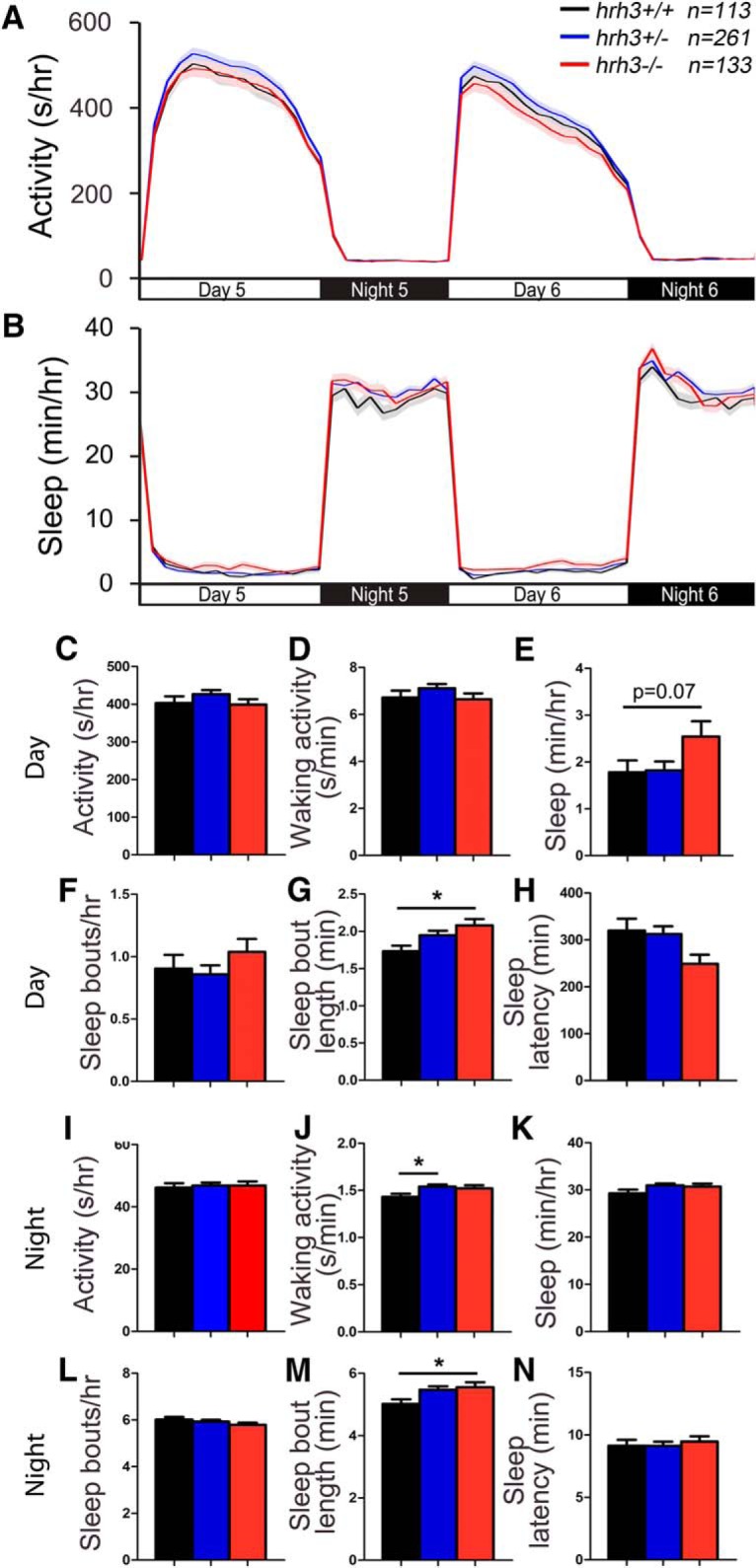
*hrh3* mutant larvae have longer sleep bouts but otherwise exhibit normal sleep/wake behaviors. *hrh3*−/− (red), *hrh3*+/− (blue), and *hrh3*+/+ (black) larvae exhibit similar amounts of all measured parameters, except that *hrh3*−/− larvae have longer sleep bouts during the day and night compared with *hrh3*+/+ siblings (***G***, ***M***), and *hrh3*+/− larvae exhibit higher waking activity at night compared with *hrh3*+/+ siblings (***J***). Line and bar graphs represent the mean ± SEM for six experiments combined; *n* indicates the number of animals analyzed. *p < 0.05 for the indicated comparisons by one-way ANOVA with Tukey’s HSD test.

Hrh3 is thought to act as an autoreceptor (Morisset et al., 2000; Arrang et al., 2007) that inhibits histamine release from histaminergic neurons, and as a presynaptic heteroreceptor on nonhistaminergic neurons to control the release of other neurotransmitters, including acetylcholine, GABA and glutamate (Schlicker et al., 1994; Haas et al., 2008; Samaranayake et al., 2016). Thus, loss of Hrh3 autoreceptors is expected to increase histamine release, and thus suppress sleep and increase wakefulness, consistent with studies using Hrh3 antagonists (Parmentier et al., 2002; Huang et al., 2006; Parmentier et al., 2007; Lin et al., 2008), but inconsistent with the hypoactivity and increased sleep observed in *hrh3*−/− mice (Toyota et al., 2002; Gondard et al., 2013). To assess the role of Hrh3 in zebrafish sleep, we generated an *hrh3* mutant that encodes for a protein that is predicted to lack two transmembrane domains and should, thus, be nonfunctional (Fig. 10). We found that *hrh3*−/− larvae have essentially normal sleep/wake behaviors compared with their *hrh3*+/− and *hrh3*+/+ siblings (Fig. 11). In contrast to *hrh3*−/− mice, which show fragmented sleep during the dark phase (Gondard et al., 2013), we found that *hrh3*−/− larvae have slightly longer sleep bouts during both the day and night (Fig. 11*G*,*M*; 11% and 12% increase during day and night, respectively, *p* < 0.05 by Tukey’s HSD test). Despite this increase in sleep bout length, the total amount of sleep in zebrafish *hrh3*−/− larvae is statistically indistinguishable from sibling controls (Fig. 11*B*,*E*,*K*), although *hrh3*−/− larvae show a trend of more daytime sleep (Fig. 11*E*) consistent with the increased nighttime sleep of nocturnal *hrh3*−/− mice (Gondard et al., 2013).

### *hrh1; hrh2a; hrh2b; hrh3* quadruple mutant larvae lack sleep-wake phenotypes

**Figure 12.**
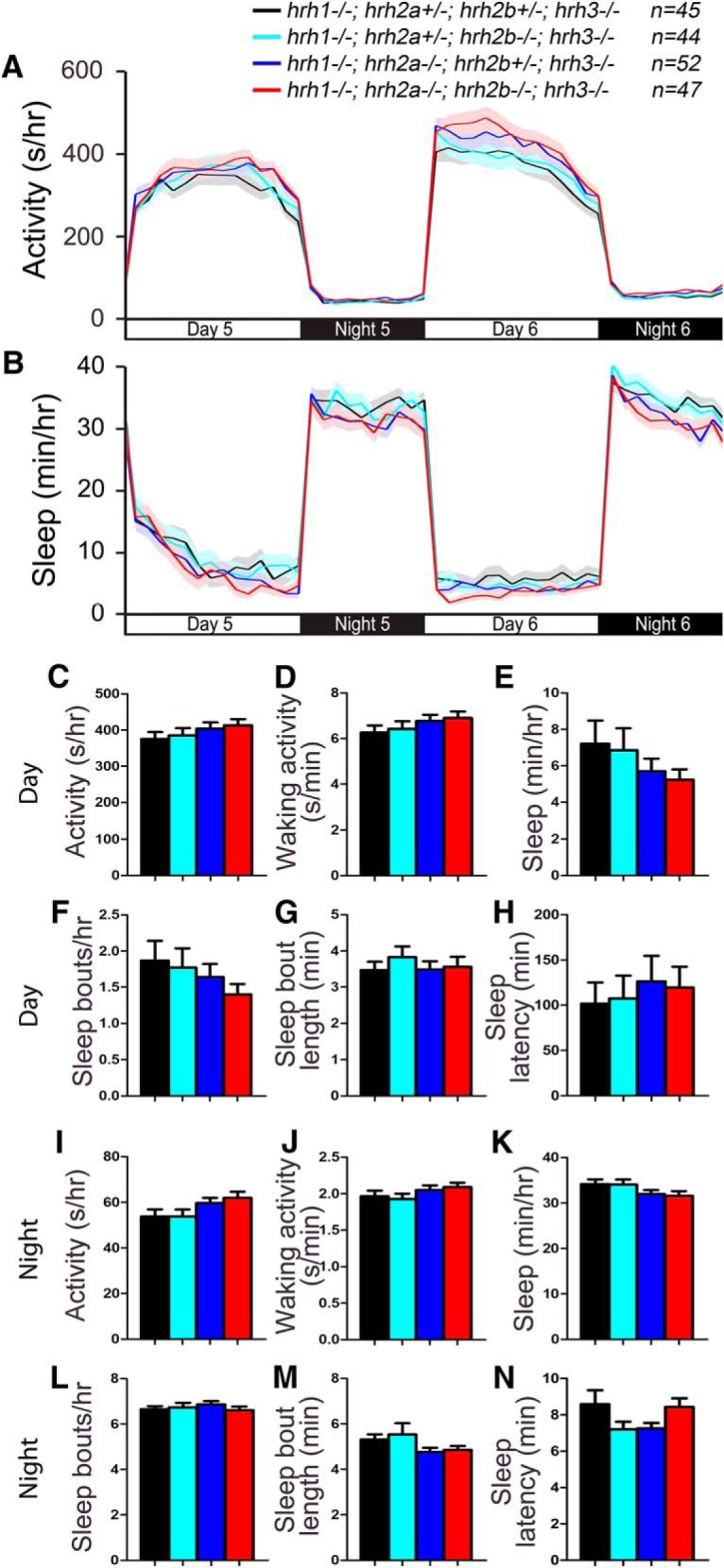
*hrh1; hrh2a; hrh2b; hrh3* quadruple mutant larvae exhibit normal sleep/wake behaviors. *hrh1*−/−*; hrh2a*−/−*; hrh2b*−/−*; hrh3*−/− (red), *hrh1*−/−*; hrh2a*+/−*; hrh2b*+/−*; hrh3*−/− (black), *hrh1*−/−*; hrh2a*+/−*; hrh2b*−/−*; hrh3*−/− (cyan), and *hrh1*−/−*; hrh2a*−/−*; hrh2b*+/−*; hrh3*−/− (blue) larvae exhibit similar amounts of all measured sleep/wake parameters. Line and bar graphs represent the mean ± SEM for two experiments combined; *n* indicates the number of animals analyzed. *p* > 0.05 for all comparisons by one-way ANOVA with Tukey’s HSD test.

To determine whether the lack of robust sleep/wake phenotypes in zebrafish Hrh mutants is due to functional redundancy, we generated and tested *hrh1; hrh2a; hrh2b; hrh3* quadruple mutant larvae. Comparison of *hrh1*−/−; *hrh2a*+/−; *hrh2b*+/−; *hrh3*−/− larvae to *hrh1*−/−; *hrh2a*−/−; *hrh2b*−/−; *hrh3−/−* siblings revealed no differences in sleep/wake behaviors (Fig. 12).

### *hdc* and *hrh1* are not required for Hcrt-induced arousal in zebrafish larvae

**Figure 13.**
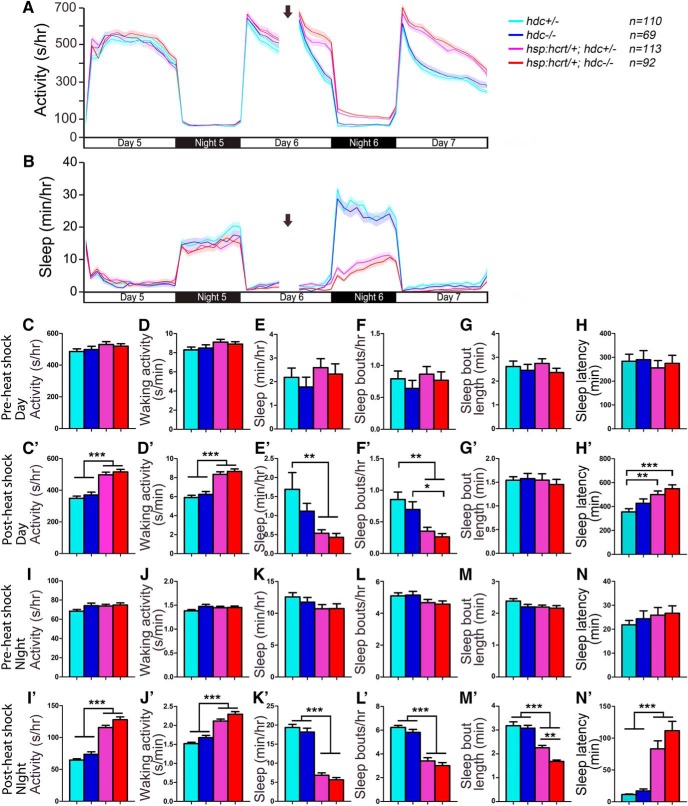
Hcrt overexpression-induced hyperactivity and reduced sleep does not require histamine. Before heat shock-induced Hcrt overexpression, there is no difference in all measured sleep/wake parameters between animals of each genotype (***A–H***, ***I–N***). Following a 1-h heat shock during the afternoon of day 6 (indicated by arrows in line graphs), during both the day and night, both *Tg(hsp:hcrt*/+*); hdc*−/− (red) and *Tg(hsp:hcrt*/+*); hdc*+/− (magenta) larvae exhibit more activity (***A***, ***C’***, ***I’***) and waking activity (***D’***, ***J’***), less sleep (***B***, ***E’***, ***K’***), fewer sleep bouts (***F’***, ***L’***), and increased latency to first sleep bout following light transitions (***H’***, ***N’***) compared with *hdc*+/− (cyan) and *hdc*−/− (blue) siblings. There is no significant difference between *Tg(hsp:hcrt*/+*); hdc*−/− and *Tg(hsp:hcrt*/+*); hdc*+/− larvae, except that *Tg(hsp:hcrt*/+*); hdc*−/− larvae have shorter sleep bouts at night than *Tg(hsp:hcrt*/+*); hdc*+/− larvae (***M’***). Line and bar graphs represent the mean ± SEM for four experiments combined; *n* indicates the number of animals analyzed. *p < 0.05; **p < 0.01; ***p < 0.001 for the indicated comparisons by two-way ANOVA with Tukey’s HSD test.

The mammalian TMN receives excitatory input from Hcrt neurons (Peyron et al., 1998; Eriksson et al., 2001; Torrealba et al., 2003; Schöne et al., 2012), and wakefulness induced by Hcrt infusion is blocked in *hrh1* knock-out mice (Huang et al., 2001) and by the Hrh1 antagonist pyrilamine in rats (Yamanaka et al., 2002; Shigemoto et al., 2004), suggesting that Hrh1 is required for Hcrt-induced arousal. However, others have shown that Hrh1 is not required for normal Hcrt function in rodents (Hondo et al., 2010), and arousal induced by optogenetic stimulation of Hcrt neurons is unaffected in *hdc* mutant mice (Carter et al., 2009). To test whether histamine is required for Hcrt-induced arousal in zebrafish, we compared the effect of Hcrt overexpression in *hdc*+/− larvae to their *hdc−/−* siblings. Consistent with a previous study (Prober et al., 2006), heat shock-induced Hcrt overexpression of *Tg(hsp:Hcrt)*; *hdc*+/− larvae increased locomotor activity and waking activity, decreased sleep and the number of sleep bouts, and increased sleep latency at night (Fig. 13). These phenotypes were statistically indistinguishable from *Tg(hsp:Hcrt)*; *hdc*−/− larvae (Fig. 13), except that *Tg(hsp:Hcrt)*; *hdc*−/− larvae had slightly shorter sleep bouts during the night after heat shock than *Tg(hsp:Hcrt)*; *hdc*+/− larvae (Fig. 13*M’* ). The latter phenotype suggests that the ability of Hcrt overexpression to decrease sleep bout length (Prober et al., 2006) is enhanced in histamine deficient animals, possibly resulting from compensatory changes in sleep/wake neuronal circuits that mediate Hcrt-induced arousal in *hdc*−/− animals. However, overall, these results suggest that *hdc* is not required for Hcrt overexpression-induced arousal in zebrafish larvae.

**Figure 14.**
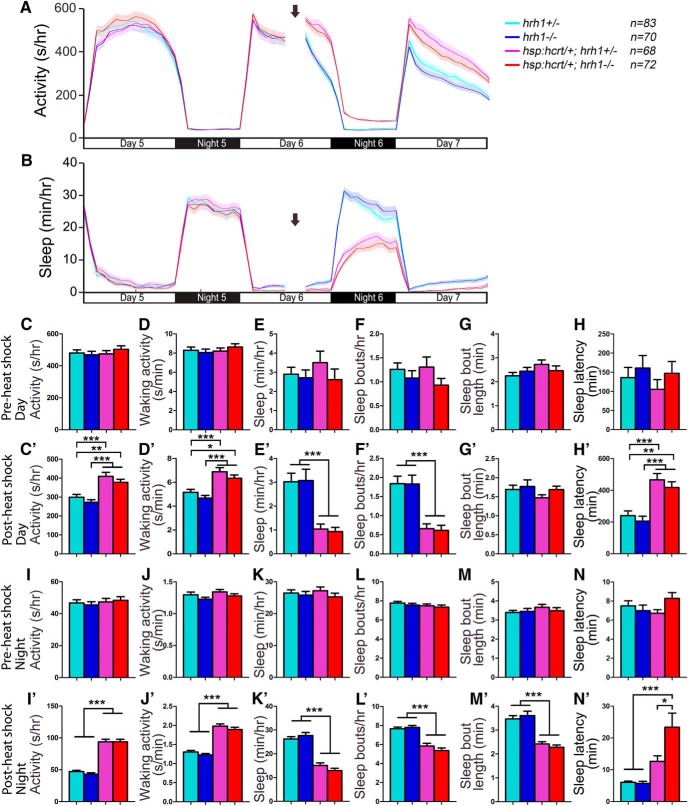
Hypocretin overexpression-induced hyperactivity and reduced sleep does not require *hrh1*. Before heat shock-induced Hcrt overexpression, there is no difference in all measured sleep/wake parameters between animals of each genotype (***A–H***, ***I–N***). Following a 1-h heat shock during the afternoon of day 6 (indicated by arrows in line graphs), during both the day and night, both *Tg(hsp:hcrt*/+*); hrh1*−/− (red) and *Tg(hsp:hcrt*/+*); hrh1*+/− (magenta) larvae exhibit more activity (***A***, ***C’***, ***I’***) and waking activity (***D’***, ***J’***), less sleep (***B***, ***E’***, ***K’***), fewer sleep bouts (***F’***, ***L’***), and increased latency to first sleep bout following light transitions (***H’***, ***N’***) compared with *hrh1*+/− (cyan) and *hrh1*−/− (blue) siblings. There is no significant difference between *Tg(hsp:hcrt*/+*); hrh1*−/− and *Tg(hsp:hcrt*/+*); hrh1*+/− larvae after heat shock, except that *Tg(hsp:hcrt*/+*); hrh1*−/− larvae have a longer latency to first sleep bout at night than their *Tg(hsp:hcrt*/+*); hrh1*+/− siblings (***N’***). Line and bar graphs represent the mean ± SEM for four experiments combined; *n* indicates the number of animals analyzed. *p < 0.05; **p < 0.01; ***p < 0.001 for the indicated comparisons by two-way ANOVA with Tukey’s HSD test.

To test whether Hrh1 is required for Hcrt-induced arousal in zebrafish, we compared the effect of Hcrt overexpression in *hrh1*+/− larvae to their *hrh1*−/− siblings. Hcrt overexpression in *Tg(hsp:Hcrt)*; *hrh1*+/− larvae increased locomotor activity and waking activity, decreased sleep and the number of sleep bouts, and increased sleep latency at night (Fig. 14). Similar to the *Tg(hsp:Hcrt)*; *hdc*−/− result, the behavior of *Tg(hsp:Hcrt)*; *hrh1*−/− larvae was indistinguishable from their *Tg(hsp:Hcrt)*; *hrh1*+/− siblings (Fig. 14), except that Hcrt overexpression failed to increase sleep latency at night in *hrh1*+/− animals but not in their *hrh1*−/− siblings (Fig. 14*N’* ). Overall, these results indicate that *hrh1* mutant zebrafish larvae exhibit normal responses to Hcrt overexpression.

**Figure 15.**
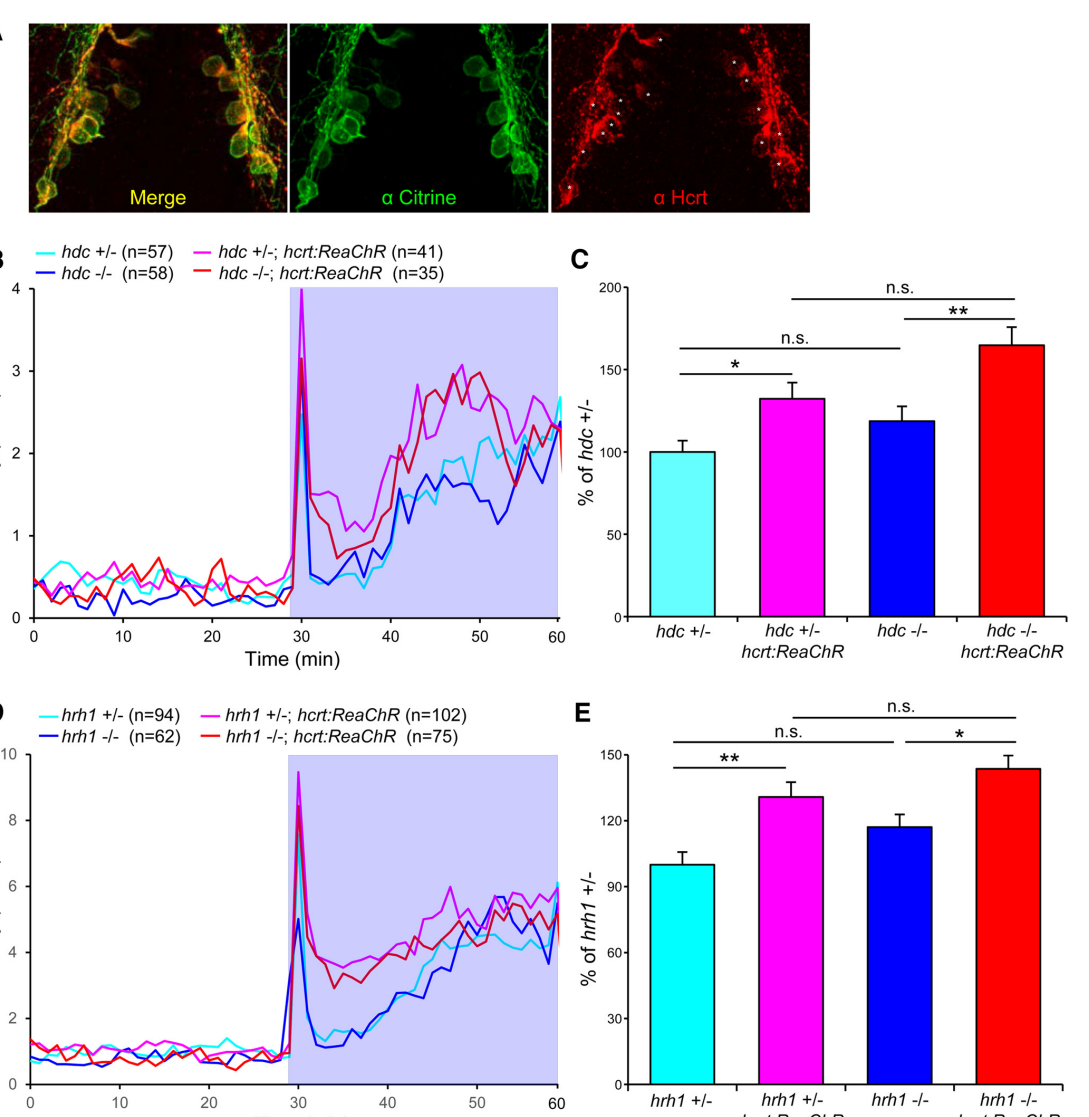
Optogenetic stimulation of *hcrt*-expressing neurons promotes locomotor activity in *hdc* and *hrh1* mutant larvae. ***A***, ReaChR-mCitrine is specifically expressed in *hcrt*-expressing neurons in *Tg(hcrt:ReaChR-mCitrine)* larvae. IHC using antibodies specific for mCitrine (green) and Hcrt (red) are shown. Asterisks indicate the soma of neurons labeled with the Hcrt-specific antibody. A maximum intensity projection of a ventral view of a 5 dpf larval brain is shown. Scale bar: 20 µm. ***B***, ***D***, Locomotor activity before (white background) and during (blue background) blue light stimulation. ***C***, ***E***, Locomotor activity during blue light stimulation normalized to average prestimulation activity level for each genotype and expressed relative to ReaChR negative siblings for *hdc*+/− (***C***) and *hrh1*+/− (***E***) larvae. Data are pooled from three (***B***, ***C***) or four (***D***, ***E***) experiments and are represented as mean ± SEM; *n* indicates the number of animals analyzed. *p < 0.05; **p < 0.01; n.s. = not significant (*p* > 0.05) by one-way ANOVA with Tukey’s HSD test.

As an alternative approach to test whether histamine signaling is required for Hcrt-induced arousal, we compared the behavioral effect of stimulating *hcrt*-expressing neurons using ReaChR (Fig. 15*A*; Lin et al., 2013) in *hdc*−/− larvae to their *hdc*+/− siblings. Similar to our previous study using *Tg(hcrt:ChR2)* (Singh et al., 2015), we found that blue light increased locomotor activity in *Tg(hcrt:ReaChR); hdc*+/− larvae compared with their *hdc*+/− siblings, as well as in *Tg(hcrt:ReaChR); hdc*−/− larvae compared with their *hdc*−/− siblings (Fig. 15*B*,*C*). Importantly, there was no significant difference in the *hcrt:ReaChR* phenotype between *hdc*+/− and *hdc*−/− animals (Fig. 15*C*). We obtained similar results for *hrh1* mutants (Fig. 15*D*,*E*). These results suggest that *hdc* and *hrh1* are not required for arousal that is induced by stimulation of *hcrt*-expressing neurons in zebrafish larvae, consistent with a rodent study (Carter et al., 2009).

**Figure 16.**
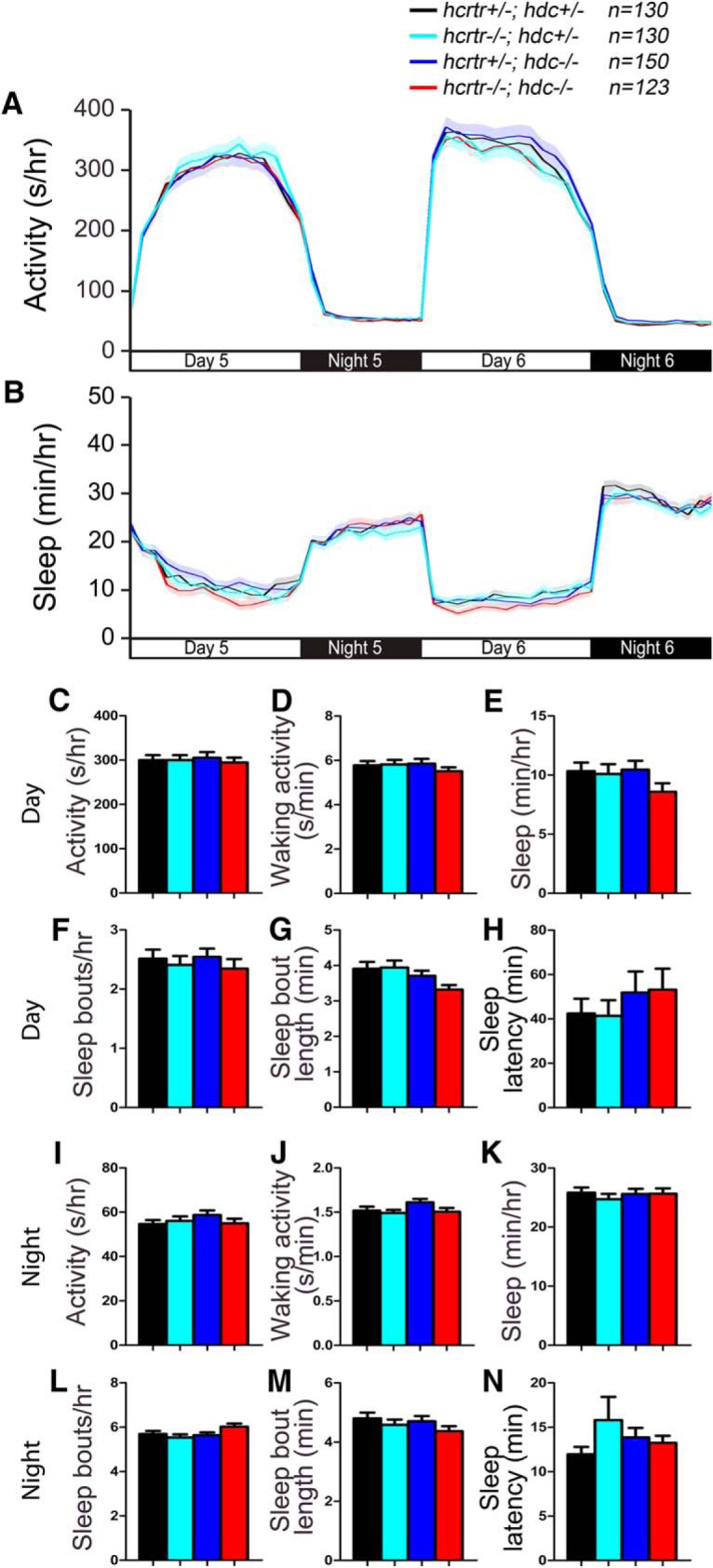
*hcrtr; hdc* double mutant larvae exhibit normal sleep/wake behaviors. *hcrtr*−/−; *hdc*−/− (red), *hcrtr*+/−; *hdc*+/− (black), *hcrtr*−/−; *hdc*+/− (cyan), and *hcrtr*+/−; *hdc*−/− (blue) larvae exhibit similar amounts of all measured sleep/wake parameters. Line and bar graphs represent the mean ± SEM for six experiments combined. Bar graphs show total values for day and night periods; *n* indicates the number of animals analyzed. *p* > 0.05 for all comparisons by one-way ANOVA with Tukey’s HSD test.

As an additional test for a functional interaction between the Hcrt and histamine systems in arousal, we examined the behavior of zebrafish larvae that lack both histamine and Hcrt signaling. Adult zebrafish with a predicted null mutation in the single zebrafish hypocretin receptor (*hcrtr*) ortholog exhibit fragmented sleep (Yokogawa et al., 2007), similar to mammals that lack Hcrt signaling (Chemelli et al., 1999; Lin et al., 1999; Peyron et al., 2000). Consistent with a previous study that failed to observe a behavioral phenotype in *hcrtr*−/− zebrafish larvae (Appelbaum et al., 2009), we observed no sleep/wake phenotypes in *hcrtr*−/−; *hdc*+/− larvae compared with their *hcrtr*+/−; *hdc*+/− siblings (Fig. 16). We also did not observe differences between *hcrtr*−/−; *hdc*+/− larvae and their *hcrtr*−/−; *hdc*−/− siblings (Fig. 16). These results suggest that the absence of sleep/wake phenotypes in *hdc* and *hcrtr* mutant zebrafish larvae is not due to functional redundancy of these two arousal-promoting systems.

**Figure 17.**
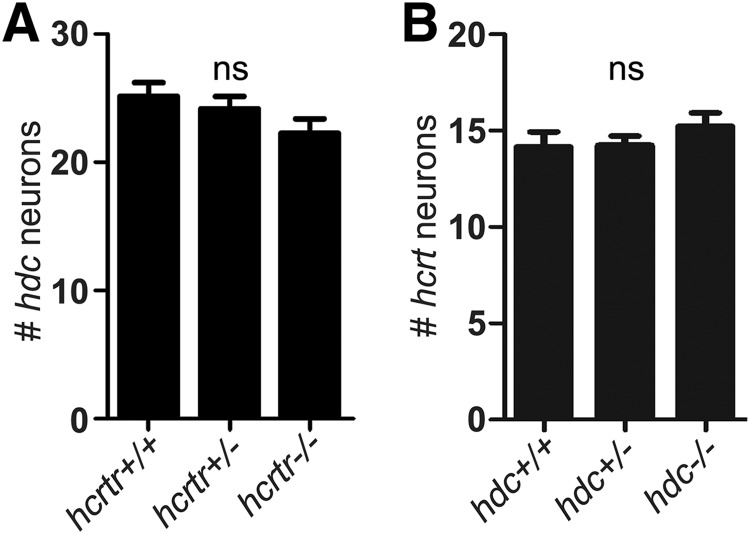
Absence of Hcrt or histamine signaling does not affect the number of histaminergic or Hcrt neurons. ***A***, There is no difference in the number of *hdc*-expressing neurons between *hcrtr*+/+, *hcrtr*+/−, and *hcrtr*−/− larvae (*n* = 21, 42, and 27, respectively). ***B***, There is no difference in the number of *hcrt*-expressing neurons between *hdc*+/+, +/−, and *hdc*−/− siblings (*n* = 13, 17, and 13, respectively). *hdc* and *hcrtr* expression was assayed by ISH at 5 dpf. ns = no statistically significant difference (*p* > 0.05) by one-way ANOVA with Tukey’s HSD test.

Narcoleptic subjects are reported to have an increased number of histaminergic neurons in the TMN compared with controls (John et al., 2013; Valko et al., 2013), whereas histamine levels are reduced in animal and human forms of narcolepsy and idiopathic hypersomnia (Nishino et al., 2001; Kanbayashi et al., 2009). In contrast to studies reporting increased histaminergic cells in narcoleptics and similar to one study of Hcrt-deficient mice (John et al., 2013), we found no difference in the number of *hdc*-expressing neurons in *hcrtr*+/+, *hcrtr*+/−, and *hcrtr*−/− sibling zebrafish larvae (Fig. 17*A*). It is possible that effects on the histaminergic system requires years of reduced Hcrt signaling, which is visible in human patients at autopsy but not in rodents sacrificed at 4-10 months or in zebrafish larvae. Alternatively, effects on histaminergic cells in human narcolepsy may result from autoimmune effects of the disease that are absent in animal models (Shan et al., 2015).

We also tested whether the absence of histamine signaling affects the number of larval zebrafish *hcrt*-expressing neurons, but we observed no difference among *hdc*+/+, *hdc*+/−, and *hdc*−/− siblings (Fig. 17*B*). This result contrasts with a previous report in which zebrafish larvae treated with an *hdc*-specific morpholino or the Hdc inhibitor α-FMH were found to have fewer *hcrt*-expressing neurons (Sundvik et al., 2011). The basis for this discrepancy is unclear, but it might result from off-target effects of the morpholino and α-FMH, or compensatory effects in the constitutive *hdc* mutant. We conclude that loss of histamine does not affect the specification of *hcrt*-expressing neurons, and loss of Hcrt signaling does not affect the specification of *hdc*-expressing neurons, in zebrafish larvae.

## Discussion

Histamine is thought to be a wake-promoting neuromodulator primarily, because administration of histamine and pharmacological inhibition of histamine degradation increases wakefulness (Lin et al., 1988; Ramesh et al., 2004), whereas pharmacological inhibition of histamine synthesis or Hrh1 signaling increases sleep (Kiyono et al., 1985; Lin et al., 1988). However, genetic studies have shown weak and often conflicting effects. For example, *hdc* mutant mice have normal sleep/wake amounts over a 24-h period (Parmentier et al., 2002) and only exhibit an arousal defect when high vigilance is required (Parmentier et al., 2002; Anaclet et al., 2009). Similarly, *hrh1* mutant mice have normal sleep/wake amounts over a 24-h period, with increased locomotor activity during the light period and slightly less activity during the dark period (Inoue et al., 1996; Huang et al., 2006).

Study of the role of histamine in mammalian sleep is complicated by its production in basophils and mast cells, where it signals via Hrh1 and Hrh2 to affect immune system function (Steinman, 2004; Haas et al., 2008; Jutel et al., 2009). Hrh1 and Hrh2 signaling can also be activated by stress via the corticotrophin-releasing hormone-mast cell-histamine axis (Elenkov et al., 2005; Tsatsoulis, 2009). The immune system has profound effects on sleep (Steinman, 2004; Opp, 2005; Rihel et al., 2010) and could account for histamine-dependent sleep phenotypes in mammals (Bollinger et al., 2009; Chikahisa et al., 2013). Indeed, inducing release of histamine from mast cells enhances wakefulness in WT mice, and the sleep-inducing effect of Hrh1 antagonists is suppressed in mast cell-deficient mice (Chikahisa et al., 2013). These results suggest that histamine produced by cells of the immune system plays an important role in arousal. In contrast, histamine is exclusively produced in the TMN in zebrafish (Da'as et al., 2011), and thus is unlikely to be involved in regulating immune system function, although this remains a possibility. In *Drosophila*, histamine is primarily produced in photoreceptors, and perturbation of histamine signaling affects the detection of light cues that regulate sleep (Melzig et al., 1996; Nässel, 1999). Thus, while a *Drosophila hdc* hypomorph exhibits increased daytime sleep (Oh et al., 2013), this may result from impaired vision.

A previous zebrafish study used a morpholino antisense oligonucleotide to knock down Hdc levels and found that these animals were less active during the day and failed to respond to a lights off stimulus (Sundvik et al., 2011). The discrepancy between our genetic results and the morpholino data could be due to morpholino-induced nonspecific toxicity that can affect behavior (Bedell et al., 2011). Indeed, 80% of morpholino-induced phenotypes are not reproduced in mutants (Kok et al., 2015), suggesting that many of these phenotypes are artifacts, although some phenotypes may be masked in mutants but not in morphants due to a poorly understood compensatory mechanism (Rossi et al., 2015). Small molecule inhibition of Hrh1 has also been reported to result in reduced locomotor activity in zebrafish larvae (Peitsaro et al., 2007; Renier et al., 2007; Rihel et al., 2010; Sundvik et al., 2011), but drugs can have off-target effects and lack the specificity of genetic perturbations. To avoid these problems, we generated zebrafish that contain predicted null mutations in *hdc* and in histamine receptors, the first such study in a diurnal vertebrate animal. There are two important caveats associated with this approach. First, while we show that *hdc* mutant zebrafish produce little or no histamine, we only have molecular evidence based on genome sequence that the histamine receptor mutations generate nonfunctional proteins. It would be preferable to show loss of histamine receptor protein using Western blotting or IHC, but antibodies specific for the zebrafish histamine receptors are not available. It is possible for a point mutation that generates a premature stop codon to be bypassed due to read-through translation (Dunn et al., 2013), and thus the zebrafish *hrh1* mutant might retain some function. However, the other zebrafish histamine receptor mutants reported in this study contain an insertion or deletion that causes a shift in translational reading frame, and thus abnormal protein sequence and several premature stop codons, before domains that are essential for protein function, including multiple transmembrane domains. It is therefore unlikely that these mutants retain any function. It would also be surprising for any of the histamine receptor mutants to have sleep/wake phenotypes given that *hdc* mutant zebrafish, which we show have little or no histamine, lack such phenotypes. Second, histamine receptors were annotated based on reciprocal BLAST searches among the human, mouse, and zebrafish genomes, which revealed a high degree of amino acid sequence conservation, and allowed unambiguous annotation of specific receptors. However, we cannot rule out the possibility that additional functionally redundant histamine receptors are present in the zebrafish genome, which could explain the absence of robust histamine receptor mutant phenotypes. Indeed, this may also be the case for rodents, which could explain why rodent histamine receptor mutants exhibit sleep/wake phenotypes that are much weaker than those observed using drugs that affect histamine signaling.

Similar to the rodent *hdc* mutant (Parmentier et al., 2002), we found that *hdc* mutant zebrafish larvae have largely normal sleep/wake behaviors. We also failed to detect major sleep/wake defects in larval zebrafish containing mutations in histamine receptors, which is generally consistent with mouse genetic studies. The murine *hrh1* knock-out displays reduced exploratory behavior in new environments but normal total locomotor activity over a 24-h period, with less slightly less activity at night but more activity during the day (Inoue et al., 1996; Yanai et al., 1998; Huang et al., 2006). We found that *hrh1* mutant zebrafish lack major defects in sleep/wake behaviors, although they are hyperactive at night, similar to *hrh1*−/− mice, which are hyperactive during the day, the rest period of these nocturnal animals (Inoue et al., 1996). Despite the lack of robust sleep phenotypes in rodent and zebrafish *hdc* and *hrh1* mutants, we do not suggest that histamine signaling has no role in arousal. Indeed, several studies have shown that acute pharmacological manipulation of histamine signaling has profound effects on arousal in zebrafish (Peitsaro et al., 2007; Renier et al., 2007; Rihel et al., 2010; Sundvik et al., 2011) and mammals (Thakkar, 2011). The most interesting potential implication of this discrepancy is that there are mechanisms that compensate for the constitutive loss of histamine signaling on arousal, possibly by strengthening of other arousal systems, which does not immediately occur in response to acute pharmacological manipulations. Consistent with this possibility, the sedating effect of the Hrh1 antagonist diphenhydramine becomes indistinguishable from placebo after only four days of treatment in humans (Richardson et al., 2002). Similar discrepancies between strong effects of acute pharmacological manipulation compared with subtle phenotypes for constitutive genetic loss-of-function have also been shown in mammals for other sleep regulatory systems, including noradrenaline and adenosine (Berridge et al., 2012; Brown et al., 2012), suggesting that this compensation may be a general feature of sleep control. Alternatively, the sedating effects of Hrh1 antagonists may be due to off-target effects. Indeed, the Hrh1 antagonist pyrilamine has local anesthetic, anti-α-adrenergic, and antimuscarinic activity, and other functions at non-Hrh1 sites (Hill and Young, 1980; Roberts and Calcutt, 1983; Nicholson et al., 1985; Leurs et al., 1995). *hrh2* is expressed sparsely and diffusely in the rodent brain, mediating inhibition in the suprachiasmatic nucleus and ventromedial hypothalamus and excitation in the dentate gyrus, hippocampus, thalamus, and cortex (Karlstedt et al., 2001; Haas et al., 2008). Studies of the effect of pharmacological inhibition of Hrh2 on sleep-wake cycles have yielded inconsistent findings. For example, the Hrh2 antagonists zolantidine and cimetidine have no effect on sleep in rats (Monti et al., 1986; Monti et al., 1990), whereas injection of the Hrh2 agonist impromidine in the preoptic region promotes wakefulness in cats. The Hrh2 antagonist ranitidine, which is used to treat stomach acid overproduction, increases feline sleep (Lin et al., 1994; Lin et al., 1996). It is unclear whether this is a direct effect of Hrh2 inhibition, or indirectly due to the alleviation of peripheral symptoms that disrupt sleep. *hrh2* knock-out mice are hypoactive (Dai et al., 2007), but sleep studies have not been described for these animals. Here, we show that *hrh2* mutant zebrafish exhibit normal sleep/wake behaviors. Thus, although Hrh2 may be involved in sleep regulation in the cat, there is no genetic evidence that Hrh2 regulates sleep in zebrafish or rodents.

Hrh3 acts as an autoreceptor to control histamine synthesis, release, and activity of histaminergic neurons (Morisset et al., 2000; Arrang et al., 2007) and as a heteroreceptor to influence the release of other neurotransmitters (Schlicker et al., 1994; Samaranayake et al., 2016). Mammalian *hrh3* is expressed in the TMN as well as in brain regions that receive histaminergic input (Haas et al., 2008). Hrh3 antagonists, such as ciproxifan and thioperamide, promote wake by suppressing cortical slow activity (0.5-8 Hz) and enhancing the frequency and amplitude of cortical fast rhythms (30-60 Hz), which is associated with higher cognitive functions (Toyota et al., 2002; Parmentier et al., 2007), and the Hrh3 antagonist pitolisant was found to reduce daytime sleepiness in 23-38% of self-reports from patients with hypersomnia (Leu-Semenescu et al., 2014). However, *hrh3* knock-out mice are hypoactive (Toyota et al., 2002; Gondard et al., 2013) and only exhibit increased wake duration during motor challenge (Gondard et al., 2013). We found that *hrh3* mutant zebrafish larvae have essentially normal sleep/wake behaviors, with slightly increased sleep bout length during the day and night. This subtle phenotype contrasts with the *hrh3* knock-out mouse, which exhibits sleep fragmentation at night (Gondard et al., 2013).

Together, our genetic studies suggest that constitutive lack of histamine signaling has minimal effects on sleep/wake behaviors in zebrafish, similar to results obtained using genetics in mice. However, mouse studies identified subtle mutant phenotypes, particularly arousal defects when high vigilance is required, such as on introduction to a novel environment, in response to changes in lighting, or motor challenge to reach palatable food (Inoue et al., 1996; Kubota et al., 2002; Parmentier et al., 2002; Abe et al., 2004; Anaclet et al., 2009; Thakkar, 2011; Gondard et al., 2013). To test whether environmental challenges might uncover behavioral defects in zebrafish histamine mutants, we subjected *hdc* and *hrh1* mutants to a mechano-acoustic stimulus and to 1-h periods of alternating light and darkness, both of which induce arousal in zebrafish larvae. These assays failed to reveal behavioral defects in either mutant, although it remains possible that other environmental perturbations or more sensitive assays might detect subtle phenotypes. Alternatively, histamine may be required to regulate arousal in adult animals and not during earlier developmental stages. This question can be addressed by studying histamine mutants in adult zebrafish and in juvenile rodents. Experiments using conditional mutants may also determine whether the essentially normal sleep/wake behaviors observed in mutants is due to compensatory mechanisms during development. It is also possible that additional proteins can act as histamine receptors but have not been targeted in our zebrafish study or in previous mouse studies. Finally, it is possible that the strong effects of acute pharmacological manipulation of histamine signaling are due to off-target effects that are independent of histamine signaling.

Genetic and optogenetic studies in rodents (Chemelli et al., 1999; Adamantidis et al., 2007) and zebrafish (Prober et al., 2006; Singh et al., 2015; Chen et al., 2016) have shown that Hcrt peptides and *hcrt*-expressing neurons play key roles in promoting arousal. Several lines of evidence suggest that the Hcrt system promotes arousal via histamine signaling in rodents. First, Hcrt neurons project to histaminergic neurons in the TMN, which express Hcrt receptors 1 and 2 (Bayer et al., 2001; Eriksson et al., 2001). Second, perfusion of Hcrt peptide into the TMN increases histamine release (Huang et al., 2001; Ishizuka et al., 2002), bath application of Hcrt excites TMN neurons in hypothalamic slices (Eriksson et al., 2001), and optogenetic stimulation of *hcrt*-expressing neurons elicits fast postsynaptic currents in histaminergic neurons (Eriksson et al., 2001; Schöne et al., 2012), suggesting that Hcrt and *hcrt*-expressing neurons can directly stimulate TMN histaminergic neurons. Third, arousal induced by Hcrt infusion is blocked in *hrh1* knock-out mice (Huang et al., 2001) and by the Hrh1 antagonist pyrilamine in rats (Yamanaka et al., 2002; Shigemoto et al., 2004), suggesting that Hrh1 signaling is required for Hcrt-induced arousal. In contrast, we found that mutation of either *hdc* or *hrh1* does not block arousal induced by Hcrt overexpression or stimulation of *hcrt*-expressing neurons in zebrafish larvae. A possible explanation for the discrepancy between larval zebrafish and adult rodents is that histamine signaling is only required for Hcrt-induced arousal in adult animals. Consistent with this possibility, *hcrt*-expressing neurons do not project to the TMN in zebrafish larvae (Prober et al., 2006), but they do so in adults (Kaslin et al., 2004). Alternatively, the requirement of Hrh1 for Hcrt-induced arousal in rodents may be due to Hrh1-mediated signaling in the immune system that is absent in zebrafish. The discrepancy between rodents and zebrafish may also reflect species-specific differences in mechanisms of Hcrt-induced arousal. However, our results are consistent with the findings that Hrh1 is not required for endogenous Hcrt signaling in rodents (Hondo et al., 2010) and that stimulation of *hcrt*-expressing neurons increases the probability of an awakening event in both WT and *hdc* mutant mice to similar extents (Carter et al., 2009). In rodents, Hcrt likely also promotes wakefulness through other arousal centers, including the noradrenergic locus coeruleus, the serotonergic dorsal raphe, and cholinergic neurons in the basal forebrain (Date et al., 1999; Bourgin et al., 2000; Eggermann et al., 2001; Liu et al., 2002; Fadel and Burk, 2010). We found that impaired noradrenergic signaling partially blocks arousal induced by Hcrt overexpression and stimulation of *hcrt*-expressing neurons in zebrafish (Singh et al., 2015), suggesting that both noradrenergic and additional arousal centers mediate Hcrt-induced arousal in zebrafish.

In summary, our genetic data suggest that histamine and its receptors are not required for normal sleep/wake behaviors in a diurnal vertebrate animal whose histamine production is limited to the brain. Our results are similar to those obtained using genetics in mice (Inoue et al., 1996; Yanai et al., 1998; Parmentier et al., 2002; Abe et al., 2004; Anaclet et al., 2009), which found only subtle sleep/wake phenotypes, and contrast with acute pharmacological manipulations of histamine synthesis and signaling, which results in strong effects on arousal in both mammals (Kiyono et al., 1985; Lin et al., 1988; Ramesh et al., 2004) and zebrafish (Peitsaro et al., 2007; Renier et al., 2007; Rihel et al., 2010; Sundvik et al., 2011). These results suggest either that mechanisms compensate for constitutive loss of histamine signaling in mutants but not in response to acute pharmacological manipulations, or that phenotypes observed using pharmacology are due to off-target effects. While it remains unclear which possibility is correct, these studies suggest that histamine signaling plays similar roles in regulating zebrafish and mammalian sleep/wake states.

## Synthesis

Reviewing Editor: Rae Silver, Columbia University Decisions are customarily a result of the Reviewing Editor and the peer reviewers coming together and discussing their recommendations until a consensus is reached. When revisions are invited, a fact-based synthesis statement explaining their decision and outlining what is needed to prepare a revision will be listed below. The following reviewer(s) agreed to reveal their identity: Paula R. Haynes, Ling Shan.

General Comments:

The authors have generated and verified constitutive loss-of-function mutants of the Zebrafish histidine decarboxylase gene. The experiments are well-thought-out, well-controlled and provide important evidence to the field of sleep research documenting differences between acute versus constitutive manipulations of arousal-promoting systems. While the outcomes, in general are negative, the findings are interesting and build a basis for further research on this topic.

In contrast to studies documenting dramatic changes in sleep/arousal in Drosophila, zebrafish, rodents and humans resulting from acute/pharmacological manipulations of the histaminergic system, the authors find that constitutive genetic loss of Hdc activity results in only very modest changes in sleep/arousal of zebrafish larvae. This is consistent with mammalian literature documenting significant, but subtle effects resulting from constitutive, genetic loss-of-function histaminergic mutations in rodents. In contrast to findings in mammals, the authors find no evidence for a genetic interaction between the Hcrt and histaminergic systems in larval zebrafish both during baseline sleep/wake behavior and during hcrt overexpression or ReaChR-induced Hcrt hyperactivation.

Although the manuscript includes data from putative histamine receptor mutants, loss of gene function has not been verified in any of these mutants. As such, these results are inconclusive and could well be excluded from the paper unless these mutants are verified to result in reduced gene function in some way. Alternatively, the authors may decide to retain the data and point directly and clearly to substantial caveats in interpreting these results, pending the availability of appropriate analytic tools.

Major concerns:

1. As noted in the general summary, none of the histamine receptor mutants have been verified to result in significantly reduced gene function. It is plausible that the lack of strong effects on arousal and/or the subtle and inconsistent phenotypes of the receptor mutants are the result of compensation by other arousal-promoting systems.

However, without further verification that these receptor mutants actually result in decreased protein levels and/or loss of receptor function these results are not interpretable and the results minimizedse figures should be removed from the manuscript. The results may be discussed briefly in the manuscript, but it must be noted that none of the mutants have been verified by other means.

Data from receptor mutants on a gene-by-gene basis may be included in the paper if the mutation is verified by further experiments to result in significantly reduced gene function. Possible verification of loss of gene function may include, but is not limited to: reduced protein expression (Western- using antibodies produced with immunogens highly homologous to putative zebrafish histamine receptors), reduced mRNA (in situ or qPCR), reduced behavioral response to receptor-specific agonists or antagonists.

2.) Small, but significant effects of genetic mutations on measured behavioral parameters are sometimes glossed over in the results where it is indicated that there is no phenotype for any of these animals. If there is an explanation whereby the significant results are best ignored, this should be discussed and explained. Otherwise, significant differences between groups should be indicated and discussed in the text, especially any results which are inconsistent with current literature indicating the histaminergic system promotes arousal. Specifically, the finding that hdc +/- larvae have decreased total sleep and reduced sleep bout length at night vs. +/+ and -/- animals should be mentioned and discussed. (Stronger homeostatic compensation in hdc -/- vs. hdc+/- animals might be a possible explanation for this.) This would also apply to receptor mutants, if they are verified and included paper- significant effects should be mentioned and discussed, not glossed over.

3. The authors concluded that the age or long-term effects are the main explanation for the discrepancy between the mouse results and human narcoleptics. However, auto-immune mechanisms (reviewed in Shan, L., Dauvilliers, Y. & Siegel, J.M. (2015) Nat Rev Neurol 11, 401-413) is another possibility.

Minor concerns:

1) The authors often overgeneralize mammalian genetic studies that found subtle phenotypes to indicate that nothing was found. When referring to mammalian experiments the authors should indicate that small, but significant phenotypes were observed and how/why this may differ from their results in zebrafish (ie. some mammalian results are specific to changes in delta power/theta power or particular sleep stages such as REM, analogs of which have not been measured in fish in this study.)

2) Figure 3- The authors conclude from these experiments that the maternal mRNA contribution is not responsible for a lack of observed sleep phenotypes, however their daytime sleep latency results do not agree with this conclusion (figure 3H vs. figure 2H) and instead suggest maternally contributed mRNA may play a modest role in daytime sleep latency, but not other sleep/arousal phenotypes. Interpretation of this experiment should be fixed in the manuscript to indicate that maternal mRNA may contribute modestly to daytime sleep latency, but otherwise does not contribute to the observed lack of sleep/arousal phenotypes in hdc-/- mutant larvae.

3) Figure 7C and F- please indicate what time period was used for this quantification ie. 12hrs during the day, 12hrs at night or 24hrs (pooled day and night).

4) Figure 15- please show quantitative comparison between hdc +/- hcrt:ReaChR and hdc -/- hcrt:ReaChR in addition to the current comparisions.

5) Though not necessarily a part of this work, the authors might find it conceptually useful to consider the relationship between sleep and the circadian system. A circadian rhythmicity of HDC expression was reported in both human and rodents (Shan, L., et al. (2012) Sleep 35, 713-715; Yu, X., et al. (2014) Curr Biol 24, 2838-2844. Genetically abolishing this rhythm, but not HDC, is the cause of altered sleep architecture in these mice.
